# Lattice-Stabilized Chromium Atoms on Ceria for N_2_O Synthesis

**DOI:** 10.1021/acscatal.3c04463

**Published:** 2023-11-28

**Authors:** Qingxin Yang, Ivan Surin, Julian Geiger, Henrik Eliasson, Mikhail Agrachev, Vita A. Kondratenko, Anna Zanina, Frank Krumeich, Gunnar Jeschke, Rolf Erni, Evgenii V. Kondratenko, Núria López, Javier Pérez-Ramírez

**Affiliations:** † Institute for Chemical and Bioengineering, Department of Chemistry and Applied Biosciences, ETH Zürich, Vladimir-Prelog-Weg 1, 8093 Zürich, Switzerland; ‡ Institute of Chemical Research of Catalonia (ICIQ-CERCA), Av. Països Catalans 16, 43007 Tarragona, Spain; § Electron Microscopy Center, Empa - Swiss Federal Laboratories for Materials Science and Technology, Überlandstrasse 129, 8600 Dübendorf, Switzerland; ∥ Laboratory of Physical Chemistry, Department of Chemistry and Applied Biosciences, ETH Zürich, Vladimir-Prelog-Weg 2, 8093 Zürich, Switzerland; ⊥ Advanced Methods for Applied Catalysis, Leibniz-Institut für Katalyse e. V., Albert Einstein-Str. 29a, 18059 Rostock, Germany; # Laboratory of Inorganic Chemistry, Department of Chemistry and Applied Biosciences, ETH Zürich, Vladimir-Prelog-Weg 1, 8093 Zürich, Switzerland

**Keywords:** single-atom catalysis, ammonia oxidation, nitrous oxide, chromium, ceria

## Abstract

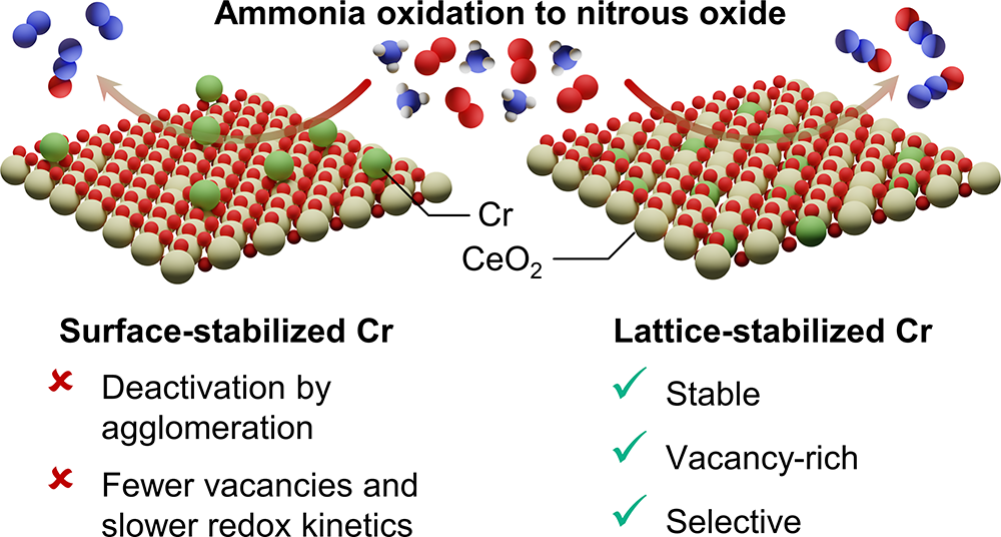

The development of selective catalysts for direct conversion of ammonia into nitrous oxide, N_2_O, will circumvent the conventional five-step manufacturing process and enable its wider utilization in oxidation catalysis. Deviating from commonly accepted catalyst design principles for this reaction, reliant on manganese oxide, we herein report an efficient system comprised of isolated chromium atoms (1 wt %) stabilized in the ceria lattice by coprecipitation. The latter, in contrast to a simple impregnation approach, ensures firm metal anchoring and results in stable and selective N_2_O production over 100 h on stream up to 79% N_2_O selectivity at full NH_3_ conversion. Raman, electron paramagnetic resonance, and in situ UV–vis spectroscopies reveal that chromium incorporation enhances the density of oxygen vacancies and the rate of their generation and healing. Accordingly, temporal analysis of products, kinetic studies, and atomistic simulations show lattice oxygen of ceria to directly participate in the reaction, establishing the cocatalytic role of the carrier. Coupled with the dynamic restructuring of chromium sites to stabilize intermediates of N_2_O formation, these factors enable catalytic performance on par with or exceeding benchmark systems. These findings demonstrate how nanoscale engineering can elevate a previously overlooked metal into a highly competitive catalyst for selective ammonia oxidation to N_2_O, paving the way toward industrial implementation.

## Introduction

Selective oxidations of hydrocarbons represent a key challenge to meet the modern standards of sustainability.^1−6^ Nitrous oxide, N_2_O, can aid in resolving it, being a highly selective oxidant able to donate a single oxygen atom while generating inert N_2_ as the sole byproduct.^7,8^ Accordingly, the issue of substrate overoxidation, frequently encountered when using O_2_, is avoided, and numerous industrially relevant reactions have been shown to proceed in fewer steps and with increased selectivity.^9−12^ However, despite the momentum garnered in the early 2000s to utilize N_2_O for chemical synthesis, its widespread adoption is still hindered by the high cost of N_2_O manufacturing, comprising five steps starting from NH_3_. Instead, propelled by the recent advances in the field of blue and green NH_3_ production, direct NH_3_ oxidation to N_2_O has emerged as a more efficient and sustainable synthesis method but requires the design of a suitable catalyst.^13−15^ In this endeavor, manganese oxide-based materials have been most widely investigated, leading to the discovery of a promising Mn–Bi–O/α-Al_2_O_3_ system at the Boreskov Institute of Catalysis.^16^ Still, issues of suboptimal N_2_O selectivity, catalyst deactivation, and the necessity to work in excess of O_2_, incurring significant downstream costs, have precluded commercialization.^17,18^ Recently, our group has reported CeO_2_-supported gold nanoparticles and low-valent manganese single atoms as highly efficient catalysts for NH_3_ oxidation to N_2_O.^19,20^ Leveraging the redox properties of CeO_2_ has enabled operation under stoichiometric conditions achieving N_2_O selectivity above 80% and 3- to 4-fold higher productivity per gram of catalyst than Mn–Bi–O/α-Al_2_O_3_. This has demonstrated that precise control over the nanostructure of the metal and the properties of the carrier are key to maximizing the catalytic effect^21,22^ and could be translated to other underexplored materials. In this respect, chromium presents an intriguing avenue of research. Owing to its capacity to assume a range of oxidation states, it has a prominent role in oxidation catalysis, such as oxidative alkane dehydrogenation^23−25^ and methane oxidation^26,27^ processes, among others. Furthermore, several recent works have reported the superior catalytic activity of chromium single-atom catalysts (SACs), such as Cr/TiO_2_ for CH_4_ oxidation^26^ or Cr/MWCNT for oxidative desulfurization.^28^ Still, very little work has been done on investigating the potential of Cr in catalyzing NH_3_ oxidation, reflected by only a few reports, primarily focused on NO formation in the high temperature (>1073 K) regime over bulk and supported chromium oxides.^29−31^ The relatively lower catalytic activity compared to manganese oxides and challenges of deactivation via irreversible reduction have led to its abandonment. However, nanoscale engineering may allow the subversion of previously encountered issues and the emergence of new catalytic synergies between the metal and the support.

Herein, we report the first effective application of chromium as a selective catalyst for NH_3_ oxidation to N_2_O, used in the form of isolated atoms embedded within the ceria lattice by coprecipitation (CP) and rivaling the performance of the state-of-the-art systems. The incorporation of a low amount of the metal into the carrier, as opposed to simple deposition on the surface, is shown to both (i) robustly anchor Cr sites, ensuring stability for 100 h on stream and (ii) facilitate the generation of oxygen vacancies, increasing their density. The Cr-induced modification of the CeO_2_ carrier further extends to the kinetics of associated redox processes, accelerating vacancy healing, as demonstrated by in situ UV–vis spectroscopy. This is complemented by the transient kinetic, spectroscopic, and computational analyses, showing the lattice oxygen of CeO_2_ to directly participate in N_2_O formation. Thus, the superior oxygen buffer capacity, coupled with the ability of Cr sites to dynamically restructure, stabilizing key intermediates of N_2_O synthesis, is identified as the central element governing catalytic performance. Our results highlight the importance of atomic precision in catalyst nanostructuring to unlock the potential of commonly overlooked metals and bring NH_3_ oxidation to N_2_O one step closer to practical application.

## Experimental Section

### Catalyst Preparation

Supported chromium catalysts on various carriers (Cr/support, support = CeO_2_, ZrO_2_, Al_2_O_3_, and Nb_2_O_5_) were synthesized via the incipient wetness impregnation (IWI) method with a nominal Cr content of 1 wt %. Accordingly, the metal precursor, Cr(NO_3_)_3_·9H_2_O (Sigma-Aldrich, 99%), was dissolved in deionized water and the resulting solution was added dropwise to the respective support, including CeO_2_ (Sigma-Aldrich, nanopowder <25 nm), m-ZrO_2_ (Alfa Aesar, catalyst support), α-Al_2_O_3_ (Alfa Aesar, 99.98%), and Nb_2_O_5_ (Sigma-Aldrich, 99.99%). After impregnation, all of the samples were dried overnight under vacuum at 353 K and then calcined in static air at 823 K (heating rate = 3 K min^–1^, hold time = 5 h).

CrCeO_*x*_-*T* catalysts (*T* = calcination temperature, i.e., 673, 873, or 1073 K) were synthesized via the CP method with a nominal Cr content of 1 wt %. Accordingly, the metal precursors, Cr(NO_3_)_3_·9H_2_O (Sigma-Aldrich, 99%) and Ce(NO_3_)_3_·6H_2_O (Sigma-Aldrich, 99%) were dissolved in 80 cm^3^ water. H_2_O_2_ (Sigma-Aldrich, 50 wt % in H_2_O) was added to oxidize the Ce^3+^ to the more easily hydrolyzable Ce^4+^ with a molar ratio *n*(H_2_O_2_)/(*n*(Ce) + *n*(Cr)) = 3 as reported elsewhere.^19,32^ NH_3_ (VWR Chemicals, 25%) was added dropwise under vigorous stirring until a pH of 9.5 was reached. The suspension was further stirred for 24 h and subsequently filtered. The residue was washed with at least 1 L of water and dried overnight in a vacuum oven at 353 K. The dried powder was calcined in static air at 673, 873, or 1073 K (heating rate of 3 K min^–1^, hold time of 5 h).

### Catalyst Characterization

Inductively coupled plasma optical emission spectrometry was conducted by using a Horiba Ultra 2 instrument equipped with a photomultiplier tube detector. The sample was dissolved in an Anton-Paar Multiwave 7000 microwave digestion system, using a 1:3 mixture of HCl (VWR Chemicals, 37%) and HNO_3_ (Sigma-Aldrich, 65%) at 513 K and 80 bar of Ar.

Powder X-ray diffraction (XRD) measurements were conducted on a Rigaku SmartLab diffractometer using Cu–Kα radiation (λ = 0.1541 nm). The data were recorded in the 2θ range of 10–70° with an angular step size of 0.017° and a counting time of 0.26 s per step.

N_2_ sorption was measured at 77 K in a Micrometrics TriStar II instrument. The sample (*m*_cat_ = 0.2 g, particle size <0.2 mm) was degassed at 473 K for 3 h prior to the analysis.

Volumetric chemisorption of O_2_ was performed at 673 K in a Micromeritics 3Flex Chemi instrument. Prior to the measurement, the sample (*m*_cat_ = 0.1 g, particle size <0.2 mm) was loaded into a U-shaped quartz microreactor, dried in He at 393 K (heating rate = 10 K min^–1^, hold time = 1 h), evacuated (hold time = 30 min), and then cooled down to 303 K under vacuum. The sample was then heated to 673 K (heating rate = 10 K min^–1^) and the O_2_ adsorption isotherms were measured.

High-angle annular dark-field scanning transmission electron microscopy (HAADF-STEM) was conducted on an aberration-corrected Hitachi HD2700CS microscope operated at 200 kV. Samples were prepared by dipping the copper grid supporting a perforated carbon foil in a suspension of the solid in ethanol and drying in air. Energy-dispersive X-ray spectroscopy (EDXS) was performed on a Thermo Fisher Scientific Talos F200X microscope with a high-brightness field emission gun operated at an acceleration potential of 200 kV. The EDXS system of this microscope is composed of 4 silicon drift detectors that enable the recording of EDXS maps with a proper signal-to-noise ratio in a relatively short collection time (5–15 min). HAADF-STEM, high-resolution scanning transmission electron microscopy (HRSTEM) images, and EDXS maps of as-prepared and used CrCeO_*x*_-673 and Cr/CeO_2_ catalysts, presented in Figure S12 of the manuscript, were acquired with a probe aberration-corrected Titan Themis operated at 300 kV, equipped with a Super-X EDX detector. The sample was prepared by dipping the copper grid supporting a perforated carbon foil in a suspension of the solid in methanol and drying it in air. The sample was cleaned gently with argon–oxygen plasma to reduce sample contamination.

X-ray photoelectron spectroscopy (XPS) was performed on a Physical Electronics Quantum 2000 spectrometer using monochromatic Al–Kα radiation, generated by an electron beam operated at 15 kV, and equipped with a hemispherical capacitor electron-energy analyzer. The sample was analyzed at an electron take-off angle of 45° and a constant analyzer pass energy of 46.95 eV with a spectra resolution step width of 0.2 eV. All XPS signals were referenced using the C 1s photoemission of adventitious carbon, which was set at 284.8 eV. Fitting of the acquired Cr 2p_3/2_ spectra was performed based on the parameters reported elsewhere, using CasaXPS software.^33^

Temperature-programmed reduction with hydrogen (H_2_-TPR) was acquired in a Micromeritics Autochem HP unit equipped with a thermal conductivity detector. The sample (*m*_cat_ = 0.3 g, particle size <0.2 mm) was loaded into a U-shaped quartz microreactor, dried in Ar at 473 K (total flow rate, *F*_T_ = 20 cm^3^ min^–1^, heating rate = 20 K min^–1^, hold time = 30 min), and then cooled to 303 K. The sample was subsequently heated to 1073 K in flowing 5 vol % H_2_ in Ar (*F*_T_ = 20 cm^3^ min^–1^, heating rate = 10 K min^–1^).

Raman spectroscopy was performed on a Horiba LabRAM HR Evolution UV–vis–NIR confocal Raman system using a Cobolt Samba Nd/YAG laser with a wavelength of 532 nm, a power of 3.2 mW and a 50× Olympus LMPlanFLN objective. Spectra were collected with an acquisition time of 10 s and an accumulation number of 5.

In situ ultraviolet–visible (UV–vis) spectroscopy was performed by using an Avantes AVASPEC fiber optical spectrometer equipped with an AvaLight-DH-S-BAL deuterium-halogen light source and a CCD array detector. The details of the UV–vis setup were described elsewhere.^34^ BaSO_4_ was used as a white reference material. The catalyst (*m*_cat_ = 0.2 g, particle size = 0.25–0.35 mm) was loaded into a quartz reactor (inner diameter = 6 mm). The catalyst bed was fixed between two layers of quartz wool. A high-temperature reflection probe, including six light fibers and one reading fiber, was positioned perpendicular to the reactor. The catalyst was heated to 673 K in flowing 10 vol % O_2_ in Ar (*F*_T_ = 20 cm^3^ min^–1^, heating rate = 10 K min^–1^, hold time = 15 min). The time-resolved UV–vis spectra (λ = 200–800 nm) were recorded every 30 s. Subsequently, the sample was flushed with Ar (hold time = 20 min), while the spectral collection was continued every 60 s. The reduction of the sample was then performed in flowing 1 vol % NH_3_ in He (*F*_T_ = 20 cm^3^ min^–1^, hold time = 30 min), followed by flushing with Ar (hold time = 20 min) and reoxidation in flowing 3 vol % O_2_ in Ar (*F*_T_ = 20 cm^3^ min^–1^, hold time = 30 min). During the reduction and reoxidation treatments, UV–vis spectra in the range of 250–800 nm and the Kubelka–Munk (KM) function at 700 nm were recorded every 5 and 1 s, respectively. *R*_rel_ was defined as a ratio of reflectance of the reduced sample, *R*_reduced_, to that of the fully oxidized one, *R*_oxidized_ (eq 1). It was vice versa for *R*_rel_ during reoxidation. From this reflectance, the relative KM function *F*(*R*_rel_) was calculated according to eq 2. The KM function was determined from the ratio of the reflectance of the oxidized sample to that of BaSO_4_:
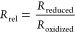
1
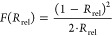
2

Temporal analysis of products (TAP) experiment was performed in the TAP-2 reactor system operating in pulse mode with a time resolution of ca. 0.100 ms.^35,36^ The catalyst (*m*_cat_ = 0.38 g, particle size = 0.15–0.4 mm) was placed between two layers of quartz particles (particle size = 0.25–0.35 mm) within the isothermal zone of an in-house-developed quartz reactor (inner diameter = 6 mm, length = 40 mm). Prior to pulse experiments, the catalyst was heated to 773 K in flowing O_2_ (*F*_T_ = 4 cm^3^ min^–1^) and kept in this flow for 0.5 h. After that it was cooled to 473 K and exposed to vacuum of ca. 10^–5^ Pa. Single-pulse experiments with ^18^O_2_/NH_3_/Ar = 1:1:1 were performed at 673 K. The total pulse size was between 7 × 10^15^ and 1.4 × 10^16^ molecules. Multipulse experiments with an NH_3_/Ar = 1:1 mixture were carried out at 673 K with the total pulse size of 7–9 × 10^15^ molecules. The feed mixtures were prepared by using NH_3_ (Messer Griesheim, 3.8), ^18^O_2_ (Campro Scientific, 97% ^18^O_2_), and Ar (Air Liquide, 5.0) without additional purification. Feed components and reaction products were quantified by an online quadrupole mass spectrometer (HAL RC 301, Hiden Analytics) at the following *m*/*z* values: 46 (N_2_^18^O), 44.0 (N_2_^16^O), 36.0 (^18^O_2_), 34.0 (^18^O^16^O), 32.0 (^16^O_2_, N^18^O), 30.0 (N^16^O), 28.0 (N_2_), 19.0 (H_2_^18^O), 17.0 (H_2_^16^O), 15.0 (NH_3_), 2.0 (H_2_), and 40.0 (Ar). During experiments with the ^18^O_2_/NH_3_/Ar = 1:1:1 mixture, the pulses were repeated 10 times for each *m*/*z* and averaged to improve the signal-to-noise ratio. In the multipulse experiments, the pulses were repeated 30 times for each *m*/*z* and treated separately without averaging. The contribution of the compounds to the respective *m*/*z* values was estimated by using standard fragmentation patterns determined in separate experiments.

Continuous-wave electron paramagnetic resonance (CW-EPR) spectroscopy experiments were conducted on a Bruker Elexsys E500 spectrometer operating at X-band frequencies, using an ER4102ST microwave resonator and equipped with an Oxford helium (ESR900) cryostat. All CW-EPR spectra were acquired at room temperature or 10 K with the following spectrometer parameters: microwave frequency = 9.8 GHz, sweep width = 590 mT, center field = 300 mT, modulation frequency = 100 kHz, modulation amplitude = 5 G, microwave power = 2.012 mW, power attenuation = 20 dB, conversion time = 1310.72 ms, and time constant = 327.68 ms. All measured *g*-factors were offset-corrected against a known standard (i.e., free radical 1,1-diphenyl-2-picrylhydrazyl).

### Catalytic Evaluation

The catalytic performance in ammonia oxidation was evaluated at atmospheric pressure in a fixed-bed microreactor (Figure S1).^19,20^ The gases, He (PanGas, purity 4.6, diluent), NH_3_ (PanGas, purity 3.8), O_2_ (PanGas, purity 5.0), and Ar (PanGas, purity 5.0, internal standard), were fed using thermal mass-flow controllers (Bronkhorst), connected to a mixing unit equipped with a pressure gauge. The catalyst [particle size = 0.15–0.4 mm, *m*_cat_ = 0.002–0.2 g for kinetic tests, and 0.05 g for stability tests; for tests at elevated GHSV (>15,000 cm^3^ h^–1^ g_cat_^–1^) the catalyst bed was diluted with SiC (particle size = 0.5–0.6 mm) to minimize the formation of hot spots] was loaded into a quartz microreactor (inner diameter = 8 mm for *m*_cat_ > 0.03 g or 2 mm for *m*_cat_ < 0.03 g), containing a bed made of quartz wool and placed in an electrical oven. The temperature of the catalyst bed was monitored and controlled using a K-type thermocouple placed in a coaxial quartz thermowell. Prior to testing, the catalyst was heated in a He flow (*T*_bed_ = 473 K, *F*_T_ = 40 cm^3^ min^–1^) for 30 min, subsequently heated to the desired temperature (*T*_bed_ = 473–723 K) and allowed to stabilize for at least 30 min before the reaction mixture (8 vol % NH_3_, 8 vol % O_2_, 4 vol % Ar, and 80 vol % He) was fed at a total volumetric flow of *F*_T_ = 50–250 cm^3^ min^–1^.

Nitrogen-containing compounds (NH_3_, N_2_, N_2_O, NO_2_, and NO), as well as O_2_ and Ar were quantified via an online gas chromatograph equipped with a GS CP-Volamine column coupled to a mass spectrometer (GC–MS, Agilent, GC 7890B, MSD 5977A). Upon acquisition of the full chromatogram, individual ion chromatograms at *m*/*z* 17, 28, 30, 32, 40, 44, and 46 were extracted. A single peak was observed on chromatograms at *m*/*z* 32, 40, and 44, allowing one to directly quantify O_2_, Ar, and N_2_O, respectively. Sufficiently different retention times of N_2_O (*t* = 1.966 s), N_2_ (*t* = 1.887 s), and NO (*t* = 1.892 s) allowed resolution of the peaks attributed to N_2_O fragments, N_2_ and NO in the product stream at *m*/*z* 28 and 30, so that subsequent quantification of N_2_ and NO could be performed. Sufficiently different retention times of NH_3_ (*t* = 1.985 s) and H_2_O (*t* = 2.065 s) allowed the resolution of their respective peaks at *m*/*z* 17 and quantification of NH_3_. No peaks were observed at *m*/*z* 46 for any of the investigated catalysts. The conversion of NH_3_ and O_2_ was calculated according to eq 3:
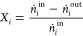
3where *ṅ*_*i*_^in^ and *ṅ*_*i*_^out^ denote the molar flows of NH_3_ or O_2_ at the reactor inlet and outlet, respectively. Selectivity toward individual products was determined according to eq 4:
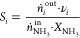
4where *v* is the number of *N* atoms in the product molecule (i.e., *v* = 2 for N_2_O or N_2_, and *v* = 1 for NO). The space-time yield (STY) of N_2_O was calculated according to eq 5:
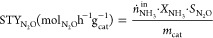
5where *m*_cat_ denotes the catalyst mass. Nitrogen (*B*_N_) and oxygen (*B*_O_) balances were evaluated for each catalytic test according to eqs 6 and 7, respectively:
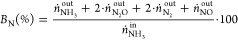
6
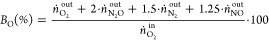
7

The error of *B*_O_ was less than 5% in all experiments. Due to the nonlinear response of the NH_3_ MS signal at low concentrations, it was adjusted accordingly to achieve a comparable value of *B*_N_ to *B*_O_. After the tests, the reactor was quenched to room temperature in a He flow and the catalyst samples were retrieved for further characterizations.

### Computational Methods

To gain insights into the possible structural configurations of isolated chromium atoms on different surfaces of CeO_2_, and their corresponding reactivity, DFT modeling was conducted with the Vienna Ab Initio Simulation Package (VASP, versions 5.4.4 and 6.3.0),^37^ using the Perdew–Burke–Ernzerhof (PBE) functional,^38^ and the HSE03 hybrid functional with 13% exact exchange (HSE03-13).^38,39^ The valence electrons were extended in plane waves with a basis set cutoff of 500 eV.^40,41^ PBE + *U* framework was employed to carry out the structural relaxations. On the Ce 4f manifolds, an additional Hubbard *U* term (*U*_eff_ = 4.5 V) was applied, following previous literature reports.^42−44^ To avoid nonphysical charge transfer caused by favored electron localization on the Ce centers due to Hubbard correction, for the substitutional structures, we also applied a value of *U*_eff_ = 4.0 V on Cr 3d. Ideally, both values should be optimized and obtained self-consistently (for instance via density functional perturbation theory),^45^ however, as they further depend on the adapted oxidation state and chemical environment of the respective center, a consistent description throughout is challenging. Thus, we deem the selected choice of parameters as a reasonable compromise between an accurate description of the complex electronic structure, and computational feasibility, in line with previous theoretical studies.^46^ Projector augmented wave (PAW) method was applied to the core electron, utilizing appropriate PAW–PBE pseudopotentials. Simulations were performed spin unrestricted, applying dipole corrections where appropriate. The threshold for electronic convergence was set at 1 × 10^–6^ eV and the positions of atoms were relaxed until residual forces reached 0.015 eV Å^–1^. For selected structures, the HSE03-13 hybrid functional was also used to refine the energies, fixing the atomic coordinates at the PBE + *U* optimized positions. In view of the significantly higher computational cost of HSE03-13, the threshold for electronic convergence was lowered to 1 × 10^–4^ eV. The “automated interactive infrastructure and database for computational science” (AiiDA) was used to drive the simulations on the high-performance computing facilities.^47−49^ Dipole corrections and spin polarization were applied throughout, while sampling of the Brillouin zone was restricted to the Gamma point.

For the (111), (110), and (100) facets of CeO_2_ slab models were constructed as (3 × 3), (2 × 2) and (3 × 3) supercells, extending 9, 6, and 9 atomic layers along the *z*-direction, of which the bottom 4, 3, and 4 layers were fixed at the optimized bulk positions, respectively. At least 10 Å of vacuum was added on top of the surfaces to minimize interactions of vertically repeated slab images under periodic boundary conditions. Formal oxidation states of chromium single-atoms (SAs) were assigned using the localized magnetic moments of reduced Ce^3+^ centers, where a threshold of 0.8 μ_B_ was applied.^50^ SA chromium adsorption and substitution energies were calculated following eqs 8 and 9, where *E*_SAC_^ads^ and *E*_SAC_^sub^ are the energies of the respective SACs, *E*_pris_ is the energy of the corresponding pristine ceria slab, *E*_Cr_ is the energy of SA chromium, using bulk Cr as reference (*E*_coh_ = −9.49 (−10.32) eV atom^–1^ with PBE + *U* (HSE03)), and *E*_Ce_ is the energy of the substituted cerium atom, evaluated via eq 10. Adsorption energies of reactants and reaction intermediates were evaluated using eq 8, accordingly:
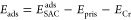
8
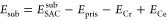
9
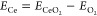
10

## Results and Discussion

### Synthesis–Performance Relationships

**Figure 1:**
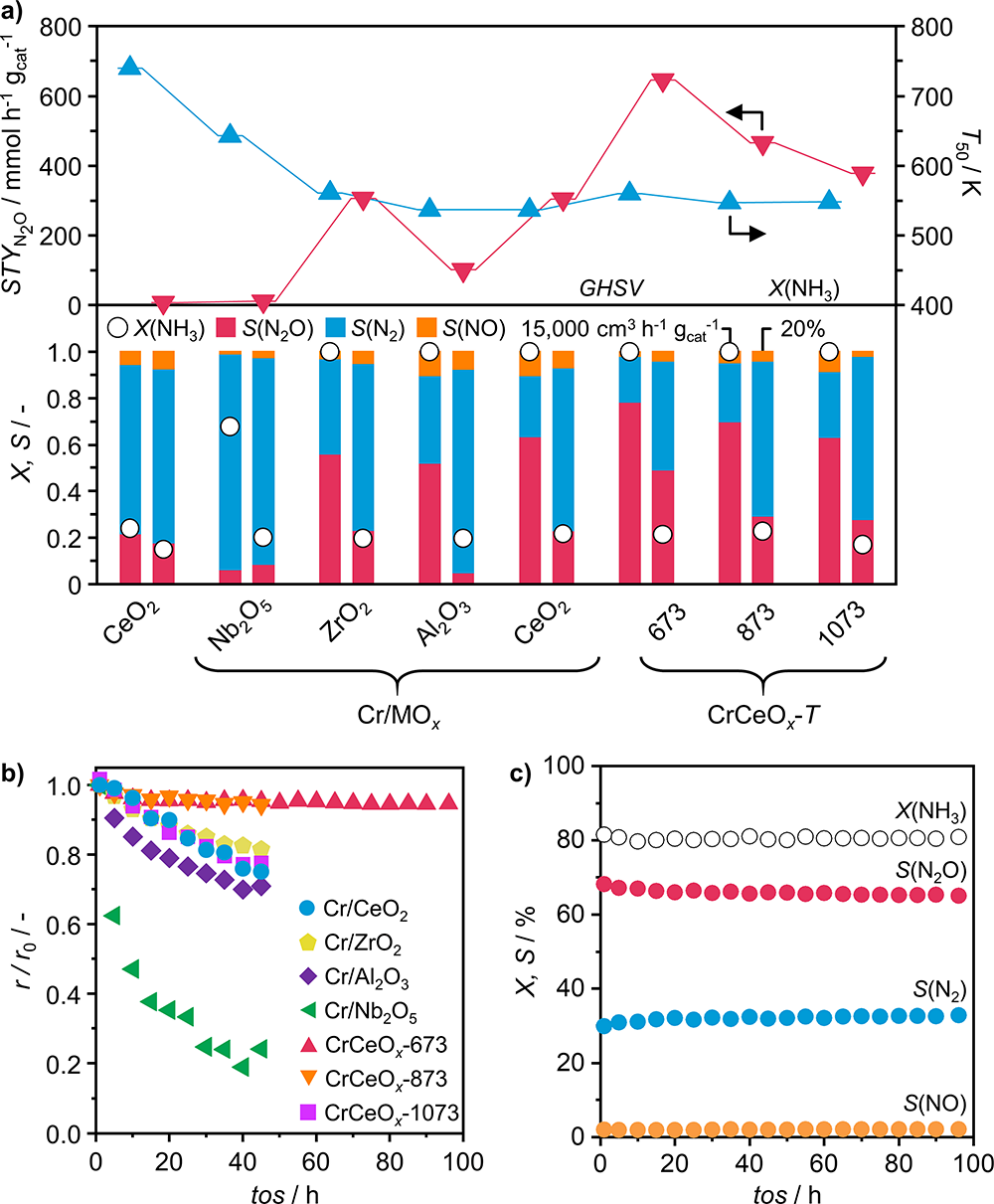
(a) Catalytic performance in NH_3_ oxidation of chromium supported on different supports (Cr/MO_*x*_), coprecipitated chromium–cerium oxides (CrCeO_*x*_-*T*), and CeO_2_ represented by the temperature at which 50% NH_3_ conversion is achieved (*T*_50_), the space-time yield (STY) of N_2_O (top panel), NH_3_ conversion and product selectivity (bottom panel). For each catalyst, the results acquired at two different gas-hourly space velocity (GHSV) values are shown, with the one on the right adjusted such that 20% NH_3_ conversion could be attained. (b) Stability test of selected catalysts expressed in terms of the normalized rate of N_2_O formation (*r*/*r*_0_), and (c) conversion and selectivity profile of CrCeO_*x*_-673 over 100 h on stream. Reaction conditions: *T*_bed_ = 673 K; *m*_cat_ = 0.002–0.2 g; GHSV = 15,000–3,000,000 cm^3^ h^–1^ g_cat_^–1^ (a), 120,000 cm^3^ h^–1^ g_cat_^–1^ (b, c); feed composition = 8 vol % NH_3_, 8 vol % O_2_, 4 vol % Ar, 80 vol % He; *P* = 1 bar.

Aiming at developing efficient Cr-based catalysts for selective NH_3_ oxidation, chromium was first deposited onto various support materials by the IWI method with a nominal Cr content of 1 wt % (Table S1, Cr/support). The catalytic performance of as-prepared materials in NH_3_ oxidation was evaluated in a fixed-bed reactor (Figure S1). First, the temperature of the catalyst bed was varied in the range of 473–723 K to investigate its effect on product selectivity and NH_3_ conversion (Figure S2). Cr/CeO_2_ displayed the highest activity, as reflected by the relatively lower temperature at which 50% NH_3_ conversion was attained (*T*_50_, Figure 1a). It also showed the highest N_2_O selectivity, reaching 64% at 623 K. To investigate whether this promotional effect of CeO_2_ could be further enhanced through closer interaction of the metal with the carrier, a set of chromium–cerium mixed oxide catalysts was prepared via a CP method, maintaining the nominal Cr content at 1 wt % and varying the calcination temperature (CrCeO_*x*_-*T*, *T* = 673, 873, or 1073 K). Notably, all of the CP samples showed similar or superior N_2_O selectivity compared to Cr/CeO_2_, with CrCeO_*x*_-673 achieving a value of 79% at 673 K (Figure 1a). The synergy between Cr and CeO_2_ was further punctuated by the fact that CeO_2_ alone is catalytically inactive. As all of the temperature ramp experiments were performed employing a low gas-hourly space velocity (GHSV = 15,000 cm^3^ h^–1^ g_cat_^–1^), it resulted in complete NH_3_ conversion at temperatures relevant for selective N_2_O formation (623–673 K). Accordingly, to assess the intrinsic catalytic activity of each system, the applied GHSV was varied (Figure S3), and the corresponding data acquired at 20% NH_3_ conversion, including the STY of N_2_O (), is shown in Figure 1a. It is immediately apparent that the product distribution of all catalysts was strongly affected by the decrease in contact time, leading to a drastic reduction in N_2_O selectivity in favor of N_2_. Nevertheless, the previously observed trend holds with CrCeO_*x*_-673 clearly standing out as the most intrinsically selective system and maintaining this feature at varying degrees of NH_3_ conversion (Figure S4). Furthermore, CrCeO_*x*_-673 also achieved the highest  of 687 mmol h^–1^ g_cat_^–1^, more than double that of its impregnated counterpart, underscoring the importance of a strong interaction between Cr and CeO_2_. Finally, the stability of each catalyst was evaluated for 50 h on stream (Figure 1b). All materials prepared by IWI, as well as CrCeO_*x*_-1073 experienced deactivation, losing between 20 and 80% of their initial activity. Conversely, the two mixed oxide catalysts calcined at a lower temperature exhibited stable performance, emphasizing the importance of additional stabilization achieved when embedding Cr within the ceria lattice. In fact, the duration of the test for CrCeO_*x*_-673 was extended to 100 h, during which both the rate of NH_3_ conversion and the corresponding product distribution remained essentially constant, firmly establishing it as a stable, selective, and highly productive material for N_2_O production via NH_3_ oxidation (Figure 1c).

### Oxygen Availability as a Performance Descriptor

Having observed stark differences between Cr-based materials in their ability to catalyze N_2_O production via NH_3_ oxidation, which appears to largely be a result of the support effect, we sought to identify the carrier property governing this behavior. Given the nature of the reaction and the variable reducibility of supports employed, redox properties were first assessed. To this end, H_2_-TPR of the catalysts and respective supports was performed (Figure S5). No reduction peak was observed for Cr/Nb_2_O_5_, which was tentatively attributed to the formation of a stable CrNbO_4_ phase.^51^ Conversely, a single reduction peak was observed for Cr/Al_2_O_3_ and Cr/ZrO_2_, at 520 and 550 K, respectively. In the former case, it likely originates from the reduction of Cr_2_O_3_ to Cr, whereas in the latter, considering the larger peak area, H_2_ likely also reacted with the surface oxygen of ZrO_2_. Similarly, in Cr/CeO_2_ a characteristic peak due to surface reduction of CeO_2_ was seen at 580 K, occurring at a lower temperature and with higher intensity than for the pristine support, suggesting that the introduction of Cr improves reducibility of CeO_2_. Among the three mixed oxide catalysts (CrCeO_*x*_-*T*), the presence of Cr also has a pronounced promotional effect on surface reducibility, with increasing calcination temperature leading to reduced H_2_ consumption and onset of reduction at a lower temperature. This is in line with variations in the surface area (*S*_BET,_Table S1), as the sample with the highest *S*_BET_ value (i.e., CrCeO_*x*_-673) has a correspondingly large reduction peak, further validating that it is attributable to the removal of surface O atoms. Furthermore, the higher reduction temperature over CrCeO_*x*_-673 serves as a fingerprint of stronger interaction between CeO_2_ and Cr in comparison with those over CrCeO_*x*_-873 and CrCeO_*x*_-1073. This is likely the result of differences in CeO_2_ crystallite size (Figure S6), which was reported to influence the metal–support interactions.^52^ Indeed, increasing the calcination temperature is expected to lead to sintering, hence resulting in larger CeO_2_ particles and lower surface area.

**Figure 2:**
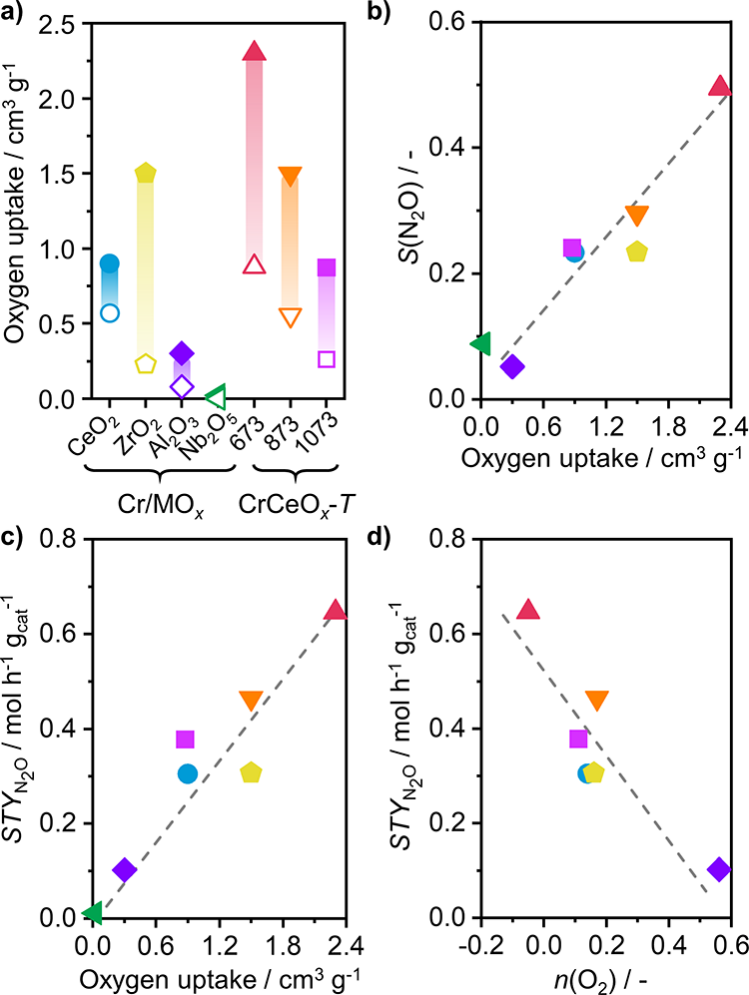
(a) Oxygen uptake of carriers (open symbols), and corresponding Cr-based catalysts (solid symbols), determined by volumetric O_2_ chemisorption, (b) N_2_O selectivity and (c)  as a function of oxygen uptake, and (d)  as a function of the partial reaction order with respect to oxygen, *n*(O_2_). Reaction conditions: *T*_bed_ = 673 K, *m*_cat_ = 0.002–0.2 g; GHSV = 15,000–3,000,000 cm^3^ h^–1^ g_cat_^–1^; feed composition = 8 vol % NH_3_, 8 vol % O_2_, 4 vol % Ar, 80 vol % He; *P* = 1 bar.

To complement the H_2_-TPR measurements and evaluate the ability of the catalysts to interact with gas-phase oxygen, we performed O_2_ chemisorption experiments at 673 K (Figure 2a). The corresponding amount of chemisorbed O_2_, herein referred to as oxygen uptake, was found to be negligible in the case of nonreducible Nb_2_O_5_ and Al_2_O_3_, while ZrO_2_, commercial and lab-synthesized CeO_2_, all exhibited appreciable interaction with O_2_. It was found to be consistently enhanced upon the introduction of Cr, while the overall ranking of catalysts remained largely unaffected, with CrCeO_*x*_-673 having the largest oxygen uptake value. It should be noted that during pretreatment of the sample, it is exposed to a vacuum, which is known to strip labile surface oxygen atoms.^53^ Thus, the measured amount of chemisorbed O_2_ also takes into account lattice oxygen that had to be replenished. Accordingly, there is good agreement between hydrogen consumption in H_2_-TPR tests and the oxygen uptake values of the catalysts (Figure S7a). Finally, to verify whether the observed differences in oxygen uptake can indeed be attributed to differences in the density of oxygen vacancies, CeO_2_-based catalysts were investigated by means of Raman spectroscopy (Figure S8a). In all samples, the Raman feature at 595 cm^–1^ could be attributed to the defect band (D-band) caused by oxygen vacancies of CeO_2_.^54,55^ The relative intensity of the D-band decreased with a rising calcination temperature for CrCeO_*x*_-*T* catalysts. The peak intensity of CrCeO_*x*_-673 and CrCeO_*x*_-873 also indicates a higher density of oxygen vacancies than that of Cr/CeO_2_, prepared by IWI. Furthermore, the D-band of Cr-free CeO_2_ materials was consistently weaker than that of their Cr-containing counterparts (Figure S8b), indicating that the introduction of Cr species induces the formation of oxygen vacancies on CeO_2_ and therefore prompts O_2_ activation. These findings are all in agreement with the above-discussed results of O_2_ chemisorption measurements. Therefore, the oxygen uptake value can serve as a robust and quantitative measure of the availability of oxygen.

When the oxygen uptake values are plotted against N_2_O selectivity, a clear trend emerges (Figure 2b). This can be understood by considering the stoichiometry of NH_3_ oxidation, which requires increasingly more oxygen to form N_2_, N_2_O, and NO. Hence, the ability to form the more oxidized species (i.e., N_2_O) is contingent upon the ample supply of oxygen, which is enabled by the use of redox-active supports, primarily CeO_2_. This point is further reinforced by the correlation between oxygen uptake and , which suggests that oxygen availability also impacts the overall rate of NH_3_ conversion—an expected outcome given that dehydrogenation of NH_3_ must be driven by the elimination of H_2_O (Figure 2c). To validate the material’s ability to modulate the oxygen supply as a key property governing catalytic performance, macrokinetic analysis was performed. Accordingly, the reaction rate was measured while varying the partial pressure of reactants and reaction temperature to extract the corresponding reaction orders (Figure S9) and the apparent activation energy (Figure S10), respectively. The obtained reaction orders with respect to O_2_ were found to have an inverse relation with , indicating that the less dependent a material is on the partial pressure of the O_2_, the higher its N_2_O productivity (Figure 2d). In fact, the best catalytic system (i.e., CrCeO_*x*_-673) exhibits a slightly negative reaction order of −0.05, which is indicative of its ability to allow the reaction to proceed via a Mars-van Krevelen (MvK) type mechanism,^56^ with the involvement of lattice oxygen, while gas-phase O_2_ could be very easily activated and has a slightly inhibiting effect. O_2_ poisoning has also been reported for Pd single atoms on defect-rich CeO_2_ nanocrystals (<8 nm) in CO oxidation.^52^ Furthermore, a similar inverse relation between  and the apparent activation energy of NH_3_ oxidation was identified (Figure S7b).

### Chromium Speciation

**Figure 3:**
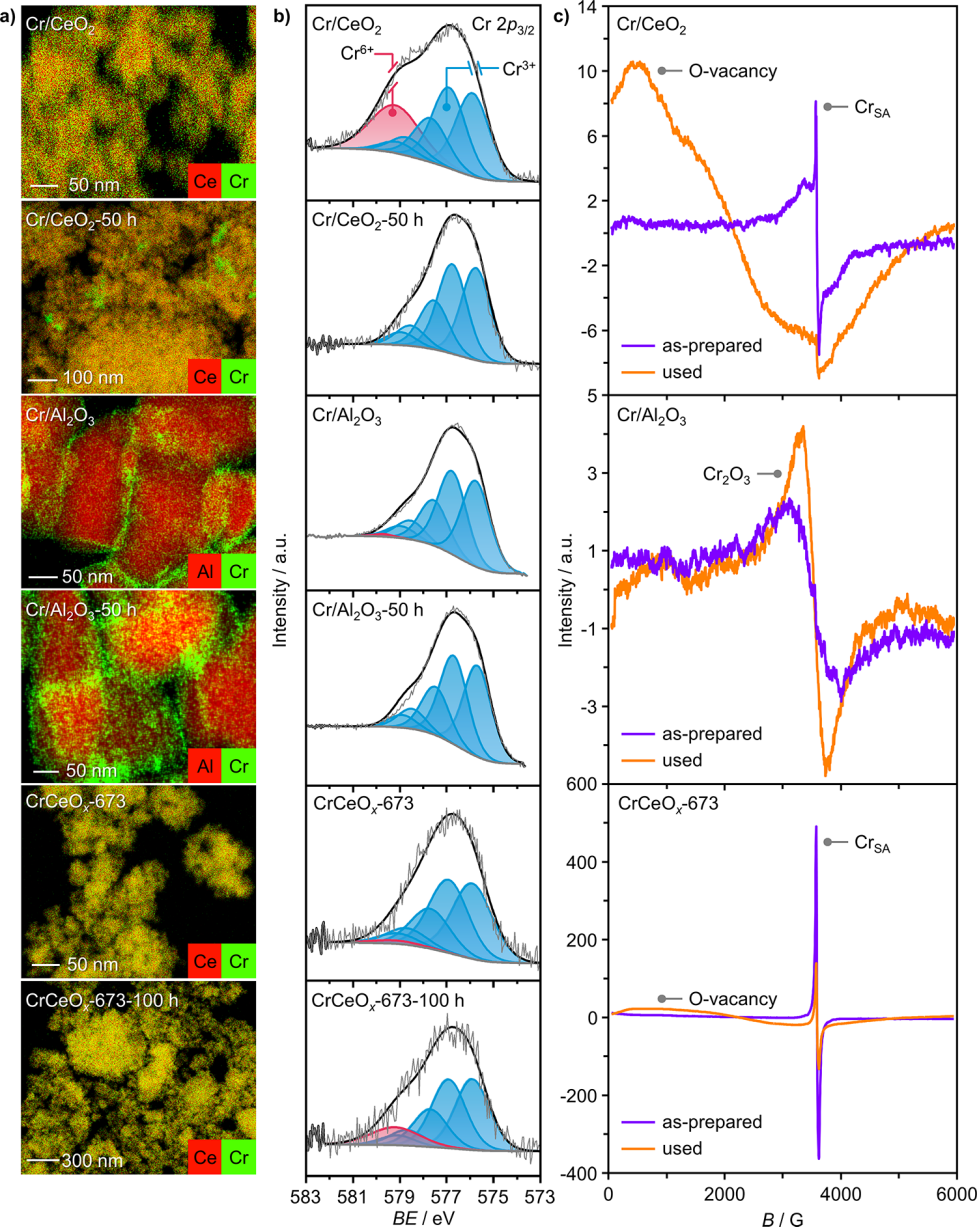
(a) STEM-EDX maps, (b) Cr 2p_3/2_ XPS spectra, and (c) EPR spectra acquired at room temperature of selected as-prepared and used Cr-based catalysts.

Having established the material’s ability to facilitate the oxygen supply as a governing descriptor of catalytic ability, we endeavored to gain a deeper understanding of underlying structural features. To shed light on the speciation of Cr, HAADF-STEM coupled to EDXS of as-prepared catalysts was performed (Figures 3a and S11). The analysis revealed that Cr is homogeneously distributed on the ZrO_2_ and CeO_2_-based systems. In fact, elemental mappings of Cr/CeO_2_ and CrCeO_*x*_-673 acquired at the nanometer scale suggest atomic dispersion of Cr. Although high-resolution HAADF-(HR)STEM could not provide direct observations of single Cr atoms, due to the relatively much larger mass of Ce, it refuted the presence of clusters or nanoparticles. Thus, since CeO_2_ (111) is the most stable facet,^57^ and was commonly observed (Figure S12), single Cr atoms are expected to predominantly reside on CeO_2_ (111). In contrast, Cr-rich regions were detected over the Al_2_O_3_ and Nb_2_O_5_ supports. Notably, despite clear evidence of nanometer-sized Cr-containing particles being present in the latter, XRD patterns of pristine supports and as-prepared Cr catalysts evidenced no differences (Figure S13). This indicates that they are either poorly crystalline or too few in number to produce characteristic reflections. To understand the origin of distinct deactivation patterns that Cr catalysts exhibited, the samples were also studied by HAADF-STEM and EDXS after the stability test (Figure 3a; Figures S11 and S12). Evidence of Cr agglomeration was found in all samples, except for CrCeO_*x*_-673 and CrCeO_*x*_-873, which are incidentally the only two to remain stable during the catalytic test. This points to Cr dispersion having a central role in ensuring and maintaining high catalytic activity. Furthermore, the contrasting behavior of Cr/CeO_2_ and CrCeO_*x*_-673 highlighted the importance of employing a suitable synthetic technique to ensure the sufficiently strong anchoring of Cr atoms and thus catalytic stability. It is also notable that while the incorporation of Cr into the lattice of CeO_2_ effectively stabilized it and prevented its agglomeration during the reaction for the samples calcined at 673 and 873 K, some agglomeration was still observed in CrCeO_*x*_-1073. A possible explanation for this behavior is the fact that this sample also showed the weakest strength of the metal–support interaction and the highest reducibility, as shown by the H_2_-TPR analysis (Figure S5). This, in tandem with a large concentration of NH_3_ in the feed and elevated reaction temperature (673 K), could have induced the migration of Cr atoms, particularly from the bulk of the catalyst, via an ex-solution-like process.^58,59^ The tendency of solid solutions to undergo such transformations is associated with the ease of surface reduction,^60,61^ which could explain why CrCeO_*x*_-673 and CrCeO_*x*_-873 did not experience a similar transformation.

Cr 2*p*_3/2_ XPS was subsequently employed to probe the electronic structure and discern the oxidation states of Cr in selected representative catalysts.^33^ The presence of both Cr^6+^ and Cr^3+^ species on the as-prepared Cr/CeO_2_ was identified, albeit with the former comprising only ∼20% of the total Cr content (Figure 3b). In contrast, only Cr^3+^ was observed on Cr/Al_2_O_3_, with Cr likely being present as the stable Cr_2_O_3_ phase (Figure 3a). Therefore, the type of support strongly affects the dispersion degree and oxidation state of the Cr species. The oxidation state of Cr in CrCeO_*x*_-673 was found to be predominantly Cr^3+^. This is likely the result of Cr occupying the positions of Ce^4+^ in the ceria lattice, forming Cr–O–Ce linkages, which inhibits further oxidation of Cr,^62,63^ and inducing the formation of oxygen vacancies, which is well in line with the high oxygen uptake of CrCeO_*x*_-673. Analysis of the used catalysts by XPS also revealed clear differences. After the reaction, Cr/CeO_2_ was comprised solely of Cr^3+^, which suggests that highly dispersed Cr^6+^ species were reduced and likely experienced agglomeration as a result, as seen in the corresponding micrographs. In contrast, the XPS spectra of as-prepared and used Cr/Al_2_O_3_ are hardly distinguishable. Similarly, the speciation of Cr in CrCeO_*x*_-673 is largely unchanged after 100 h on stream, with only a minor increase in Cr^6+^, perhaps as a result of the formation of a surface chromate species.

To deepen the understanding of Cr speciation, electron paramagnetic resonance (EPR) spectroscopy was utilized. The continuous-wave (CW) EPR spectrum of the as-prepared CrCeO_*x*_-673 acquired at room temperature was found to mainly consist of a narrow signal around *g* = 2, characterized by an anisotropic *g* tensor (Figure 3c). This signal could be attributed to low-spin Cr^3+^ (*d*^3^, *S* = 1/2) centers, corresponding to magnetically isolated ions in a highly distorted orthorhombic coordination. This indicates that Cr is highly dispersed and likely incorporated inside the ceria lattice. Additional minor signals are present and partially overlap with the main signal. These can be attributed to Cr^5+^ and/or Ce^3+^. Measurements at 10 K showed the presence of an additional weak low-field signal around *g* = 4 (Figure S14), characteristic of strongly axially distorted *S* = 3/2 centers with zero-field splitting, higher than the Zeeman interaction. This signal could be attributed to high-spin Cr^3+^ (*d*^3^, *S* = 3/2) in a more symmetric, tetragonally distorted environment, possibly coordinated on the surface. The Cr/CeO_2_ sample showed the same, yet significantly weaker, low-spin Cr^3+^ “single-atom” signal that was already observed for the CP sample (Figure 3c). The narrow Ce^3+^/Cr^5+^ signal is also observed. In addition, partially overlapping with the latter, a broader unstructured signal is present, which is most likely due to dipolar or exchange-coupled Cr^3+^ ions and may be attributed to small Cr_2_O_3_ clusters and aggregates. Furthermore, the overall intensity of the Cr^3+^ signals is significantly lower compared to CrCeO_*x*_-673, suggesting that a large amount of Cr^3+^ is present as EPR-silent antiferromagnetically coupled dimers. The low-temperature spectrum (Figure S14) also showed a *g* = 4 feature, attributed to high-spin Cr^3+^ in tetragonal coordination. Moreover, a series of broad peaks could be observed, centered around *g* = 2 and with an average spacing of approximately 800 G (Figure S14). This pattern could be attributed to the fine splitting due to spin–orbit coupling in a high-spin *S* = 3/2 system with zero-field splitting, comparable to the Zeeman interaction (and consequently lower than for the CP sample). The local geometry of surface-coordinated Cr^3+^ in Cr/CeO_2_ is therefore very different from CrCeO_*x*_-673, being less axially distorted and closer to orthorhombic. Finally, the Cr/Al_2_O_3_ sample exhibited a distinct, unstructured cluster signal, similar to the one observed for the Cr/CeO_2_ sample, showing that Cr is highly aggregated on the Al_2_O_3_ surface, most likely in the form of Cr_2_O_3_ clusters and nanoparticles.

When considering the room-temperature spectra of the used samples (Figure 3c), the low-spin Cr^3+^ signal in CrCeO_*x*_-673 decreased by about 60%, while its line shape remained unchanged. This could be partially attributed to Cr^3+^ oxidation to the EPR-silent Cr^6+^, evidenced by XPS. At the same time, the intensity of the high-spin Cr^3+^, appearing at low field and observable at low temperature, is virtually unchanged. In addition, the spectrum shows extremely broad and strongly anisotropic signals, covering the whole experimental field sweep. This signal exhibits the typical characteristics of extended ferro/antiferromagnetically coupled systems, closely resembling the signals attributed to exchange-coupled oxygen vacancy-bound polarons, previously observed in oxygen-depleted catalysts, based on In_2_O_3_ and ZrO_2_.^64^ Similarly, the spectrum of used Cr/CeO_2_ exhibited a broad oxygen vacancy-related signal. However, its intensity is significantly lower, which agrees with its lower oxygen uptake value. Furthermore, the intensity of the sharp “single-atom” signal is drastically reduced, which is in line with the agglomeration of Cr observed in EDXS mappings (Figure 3a). Any signal from Cr^3+^ ions in formed Cr_2_O_3_ agglomerates is expected to be broad as well and cannot be separated from the broad oxygen vacancy-related signal. Conversely, the spectrum of the used Cr/Al_2_O_3_ still evidenced the signal characteristic of Cr_2_O_3_ clusters and nanoparticles, showing little difference from the spectrum of the as-prepared material (Figure 3c). No oxygen vacancy-related signals were observed, indicating that lattice oxygen did not participate in the reaction, which is consistent with the nonreducible nature of Al_2_O_3_.

### Catalytic Role of Lattice Oxygen

**Figure 4:**
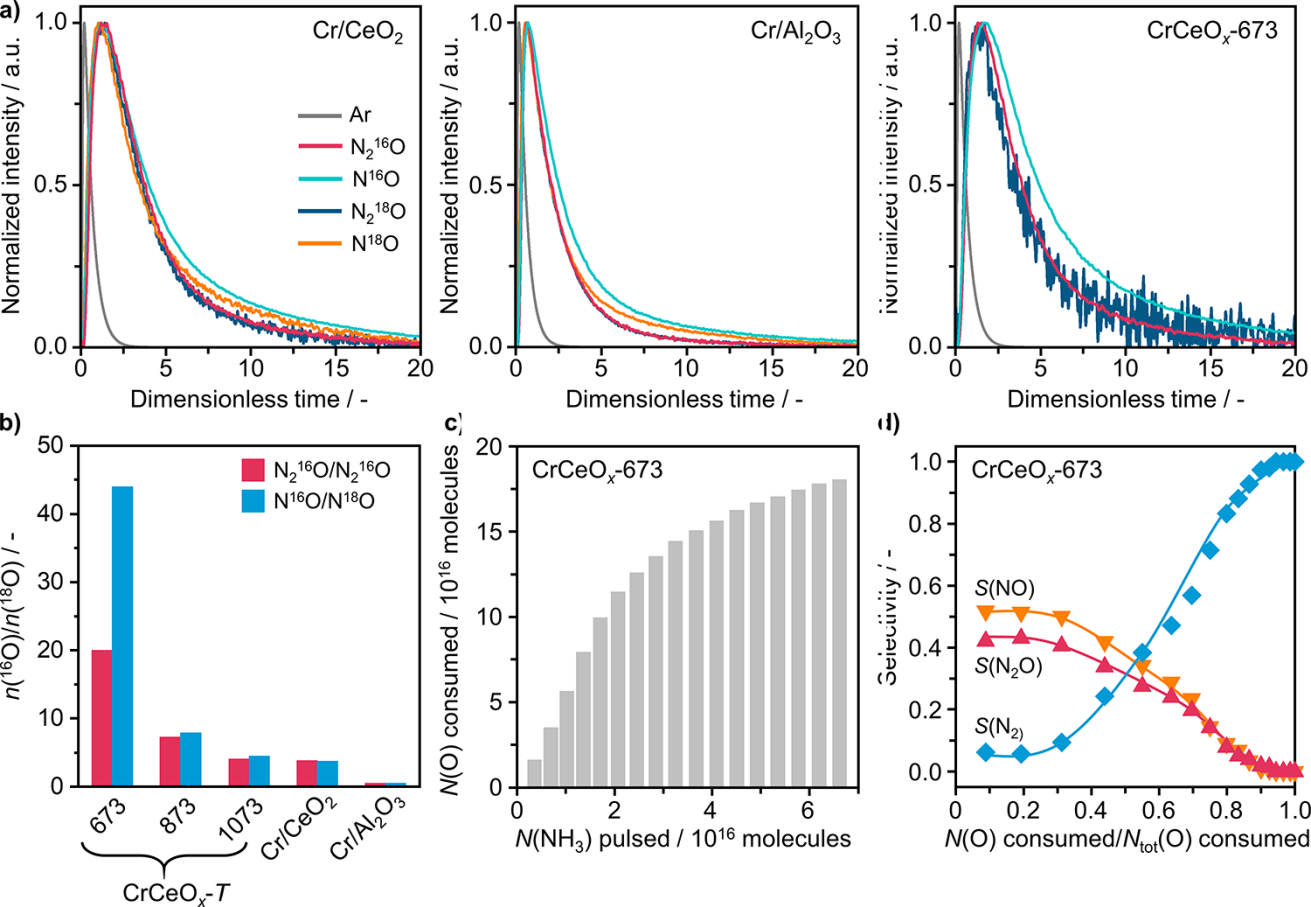
Temporal analysis of products (TAP) of NH_3_ oxidation over selected catalysts. (a) Height-normalized transient responses in NH_3_ oxidation, and (b) relative contribution of lattice (^16^O) and gas-phase (^18^O) oxygen to N_2_O and NO formation, expressed as molar ratio of products, after pulsing ^18^O_2_/NH_3_/Ar = 1:1:1 at 673 K. The transient response of N^18^O over the CrCeO_*x*_-673 catalyst is not shown due to its small quantity and strong noise. (c) Amount of lattice oxygen removed from CrCeO_*x*_-673, and (d) corresponding product selectivity upon successive pulsing of NH_3_/Ar = 1:1 at 673 K over this catalyst.

With availability of lattice oxygen established as a performance descriptor, its direct participation in NH_3_ oxidation to N_2_O was studied by using a TAP reactor.^36,65^ Initially, pulse experiments with a mixture of ^18^O_2_/NH_3_/Ar = 1:1:1 at 673 K were performed. The use of isotopically labeled ^18^O_2_ enabled us to discern the origin of O in N_2_O from either lattice of CeO_2_ or adsorbed O_2_ from the gas phase. The transient responses of the products are shown in Figure 4a. Quantitative analysis revealed that the abundance of ^16^O-containing products depends strongly on the catalyst used (Figure 4b). In the case of CrCeO_*x*_-*T*, the ratio of ^16^O- and ^18^O-containing products for both N_2_O (N_2_^16^O/N_2_^18^O) and NO (N^16^O/N^18^O) decreased with the increasing of calcination temperature of the catalyst and hence diminishing density of oxygen vacancies. Remarkably, these two ratios over CrCeO_*x*_-673 exceed 20 and 40, respectively, indicating that N_2_^16^O and N^16^O constitute over 95% of O-containing products. Cr/CeO_2_ also showed a N_2_^16^O/N_2_^18^O and N^16^O/N^18^O ratio exceeding 1 (ca. 3.8). These results clearly prove the direct participation of lattice oxygen (^16^O) of CeO_2_ in the formation of N_2_O and NO, which is in line with the MvK mechanism proposed earlier. Conversely, gas-phase O_2_ is primarily responsible for the replenishment of oxygen vacancies. This is supported by the identical shape of transient responses of N_2_^16^O and N_2_^18^O, as well as of N^16^O and N^18^O. In contrast, only minor fractions of N_2_^16^O and N^16^O were detected over Cr/Al_2_O_3_ (Figure 4b). This can be attributed to the nonreducible nature of Al_2_O_3_. To investigate the effect of CeO_2_ reduction degree on product formation, a multipulse experiment with a mixture of NH_3_/Ar = 1:1 was performed at 673 K over CrCeO_*x*_-673. With the increase in the number of NH_3_ pulses, the progressive depletion of lattice oxygen in the catalyst could be detected (Figure 4c). Moreover, the change in the reduction degree was found to influence the product selectivity (Figure 4d). It was essentially constant until 20% of available oxygen was depleted, producing almost exclusively N_2_O and NO. This buffer region is indicative of the ample supply of oxygen provided by the catalyst. With the gradual consumption of lattice oxygen, the selectivity to N_2_O and NO decreased, whereas the selectivity to less oxidized N_2_ increased. These results complement the conclusion drawn from the single-pulse experiments (Figure 4a,b) and clearly demonstrate that NH_3_ oxidation proceeds via a MvK mechanism over CeO_2_-based catalysts.

**Figure 5:**
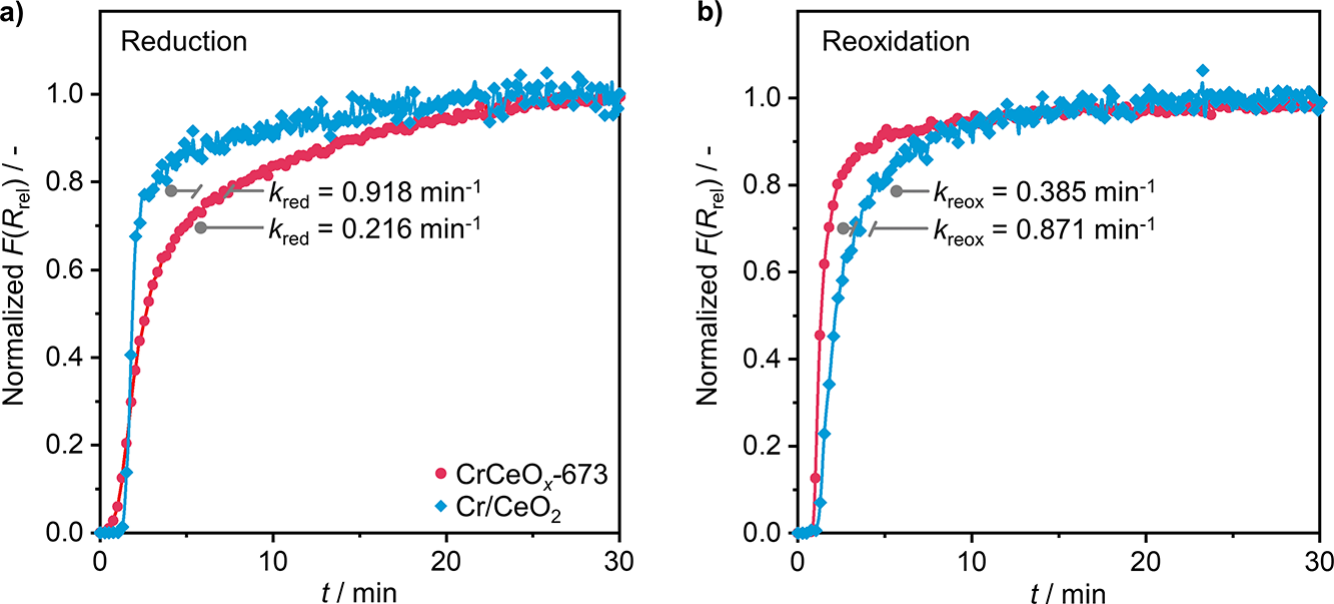
Normalized temporal changes in the relative Kubelka–Munk function at 700 nm during in situ (a) reduction and (b) reoxidation treatments of CrCeO_*x*_*-*673 and Cr/CeO_2_. The corresponding UV–vis spectra are shown in Figure S15. The rate constants of reduction and reoxidation were acquired by linear fitting in the region of steepest increase, only considering the data points that yield a linear correlation with *R*^2^ > 0.9. Reaction conditions: *T* = 673 K; *m*_cat_ = 0.2 g; *F*_T_ = 20 cm^3^ min^–1^; feed composition: 1 vol % NH_3_ in He (reduction); 3 vol % O_2_ in Ar (reoxidation); and *P* = 1 bar.

The dynamic redox cycle involved in the MvK mechanism, comprising the consumption of lattice oxygen and healing of generated vacancies, requires facile activation of gas-phase O_2_ to sustain the availability of active oxygen species. To study the kinetics of the oxidation and reduction processes, time-resolved in situ UV–vis spectroscopy was used. For this purpose, the as-prepared Cr/CeO_2_ and CrCeO_*x*_-673 were first fully oxidized in 10 vol % O_2_/Ar, then reduced by 1 vol % NH_3_/He and consecutively reoxidized by 3 vol % O_2_/Ar at 673 K. At the same time, UV–vis spectra were recorded to monitor the catalyst state (Figure S15). Upon exposure to NH_3_, the intensity in the UV–vis spectrum above 500 nm increased due to the reduction of the catalyst.^66^ When no further changes to the spectrum were observed, the feed composition was changed, and the UV–vis spectrum was acquired during the reoxidation process, until the initial state of the catalyst was recovered, signifying that the redox cycle was reversible. The normalized temporal changes in the KM function at 700 nm were subsequently used to evaluate the redox behavior of the catalyst during reduction and reoxidation (Figure 5). As indicated, the rate of surface reduction was found to be about four-fold higher over Cr/CeO_2_ than over CrCeO_*x*_-673, whereas reoxidation proceeded twice as fast over CrCeO_*x*_-673 (Figure 5). These observations suggest that under the typical conditions employed for NH_3_ oxidation, where the reducing (i.e., NH_3_) and oxidizing (i.e., O_2_) agents are present in stoichiometric amounts, CrCeO_*x*_-673 should generally remain in a more oxidized state. Thus, the fast redox kinetics coupled to the larger pool of available lattice oxygen significantly enhances the oxygen buffer ability of CrCeO_*x*_-673, which we put forward as the reason for the superior N_2_O selectivity and productivity of this catalyst.

### Experimentally Guided Modeling of N_2_O Formation

**Figure 6:**
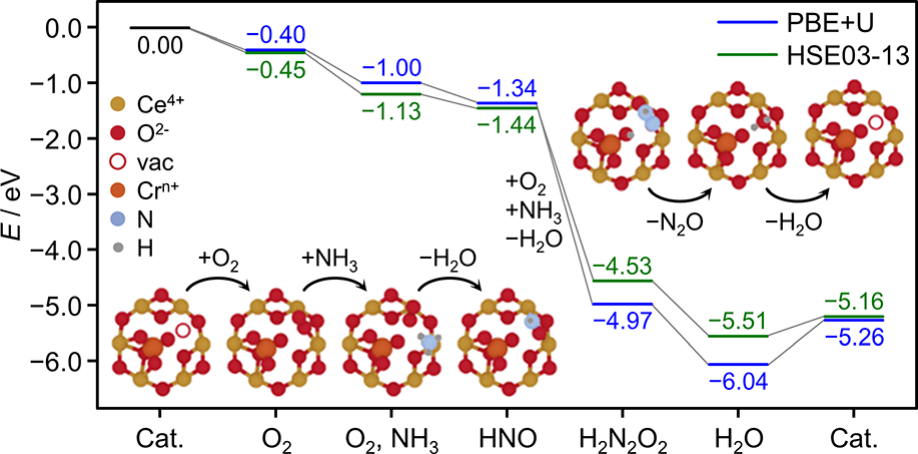
Feasible reaction pathway proceeding via the proposed nitroxyl, HNO, and hyponitrous acid-like, H_2_N_2_O_2_, intermediates en route to N_2_O formation is shown for a representative CrCeO_*x*_ catalyst model based on the majority CeO_2_ (111) facet. Energies (in eV) were evaluated with PBE + *U* and the HSE03-13 hybrid functionals (also see Table S4).

Having established that the CeO_2_ carrier is the primary source of active oxygen species for the reaction, we aimed to expand our understanding of the mechanism by density functional theory (DFT) simulations. As a starting point, suitable models for the impregnated Cr/CeO_2_ and coprecipitated CrCeO_*x*_ catalysts had to be developed. It should be noted that although EPR analysis suggested that some Cr in the Cr/CeO_2_ sample might be present in the form of antiferromagnetically coupled Cr^3+^ dimers, as well as a small amount of Cr_2_O_3_ aggregates, the metal still appears to primarily occur as isolated Cr^3+^ sites. Thus, we constructed a catalyst library of possible adsorbed and substitutional Cr SA structures based on the CeO_2_ low-index facets to model Cr/CeO_2_ and CrCeO_*x*_-673 catalysts, respectively (Figure S16). In line with the observation that Cr/CeO_2_ is not stable under the reaction conditions, we identify adsorbed SA Cr on the CeO_2_ (111) facet as only a metastable state (Figure S16 and Table S2), contrary to the favorable formation of substitutional CrCeO_*x*_ (Table S3). Here, replacing a surface Ce center by Cr leads to an initial threefold surface oxygen coordination, which restructures during optimization due to the much smaller size of Cr compared to Ce. In the resulting structure, the symmetry is lowered and the Cr center adopts a tetrahedral coordination sphere, reminiscent of the chromate ion, CrO_4_,^2^ as shown in Figures 6 and S16. Importantly, despite one of the oxygen ligands of Cr being removed from its original lattice position, breaking two Ce–O bonds, and introducing a formal vacancy in the process, Cr retains the 4+ oxidation state of the replaced Ce^4+^ (Table S3). However, when an actual surface oxygen vacancy of the ceria lattice, away from the SA center, is introduced (Figure S17), the Cr atom gets reduced to a 3+ state, in line with previous reports for the bulk substitutional position.^63^ Thus, under vacuum conditions present during XPS measurements, oxygen expulsion and surface reduction likely lead to substitutional Cr^3+^ as the major species.

We also found that upon removal of the dangling Cr-bound oxygen ligand (which might, for instance, be consumed during initial NH_3_ dehydrogenation), another restructuring occurs, in which Cr restores its chromate-like tetrahedral coordination sphere by moving further down into the lattice and forming bonds with subsurface oxygen centers (Figure S17). Accordingly, this restructuring leads to a second surface oxygen vacancy in the lattice with a formation energy of 2.11 eV (0.72 eV) when evaluated with HSE03-13 (PBE + *U*), comparable with (significantly lower than) vacancy formation energies previously reported for the pristine CeO_2_ surface.^67^ Thus, integration of Cr into the lattice not only facilitates vacancy formation of the CeO_2_ support but also unlocks an alternate mechanism of providing active surface oxygen species.

Next, we evaluated the adsorption of the reactants, namely, O_2_ and NH_3_, and the formation of the HNO and H_2_N_2_O_2_ intermediates, considering Cr of the (111)-based adsorbed Cr/CeO_2_ catalyst, as well as Cr and Ce centers of the adjacent vacancy of the restructured, substitutional CrCeO_*x*_ catalyst as possible adsorption sites. We began our exploration of the reactivity landscape with the mechanistic scheme proposed for the previously reported CeO_2_-based Mn catalyst,^20^ where the reaction sequence begins by the adsorption and activation of gas-phase O_2_, followed by dehydrogenation of NH_3_ to yield nitroxyl, HNO, a key intermediate en route to N_2_O formation. Then, a second HNO is generated accordingly, and N–N bond formation is achieved by the dimerization of both fragments mediated by the metal center. This results in a hyponitrous acid-like intermediate, H_2_N_2_O_2_, which is stabilized via ring formation with the low-valent Mn atom. Its dissociation finally leads to the elimination of N_2_O and H_2_O, recovering the catalyst.

Interestingly, in the substitutional (111)-based CrCeO_*x*_ system, we found that the Cr atom cannot bind the reactants due to its coordinately saturated state. Instead, adsorption on Ce of the adjacent vacancy is strongly favored (Table S4). We therefore deem it likely to be the reactive site of the catalyst. The structures of the main intermediates along with the energies of the reaction sequence based on this site are presented in Figure 6. Initial adsorption of O_2_ is more favorable than adsorption of NH_3_ (Table S4), thus it is assumed to occur first (and be responsible for the slightly negative reaction order with respect to O_2_). Importantly, the second Ce atom remains accessible for the coordination of NH_3_, bringing both reactants into the proximity and facilitating the dehydrogenation of NH_3_. The resulting HNO fragment is then stabilized in the oxygen vacancy. After the second dehydrogenation, however, the proposed hyponitrous acid-like ring intermediate is not stable in its fully protonated state. Proton transfer to the dangling Cr-bound oxygen atom, as shown in Figure 6 (or, alternatively, the CeO_2_ surface), instead allows for a stable ring intermediate. The elimination of N_2_O and H_2_O finally ended the cycle. The analogous reaction sequence for the Cr/CeO_2_ system (Figure S18) rationalizes that adsorbed low-valent Cr can initially show reactivity similar to CrCeO_*x*_. However, as the isolated Cr is not stable in this structure, catalytic activity is lost over time on stream due to agglomeration.

To summarize, the integration of Cr into the lattice via CP naturally facilitates the formation of oxygen vacancies, in line with the increased oxygen uptake of the system, while the incurred structural distortions activate the surrounding CeO_2_ surface, allowing for the reaction to proceed. Thus, our computational investigation again points to the fundamental role of the availability of reactive oxygen for the formation of N_2_O. Lastly, as seen in the hyponitrous acid-like intermediate, the Cr-bound oxygen can also participate in surface acid–base equilibria, possibly assisting reactivity.

### Catalyst Benchmarking

**Figure 7:**
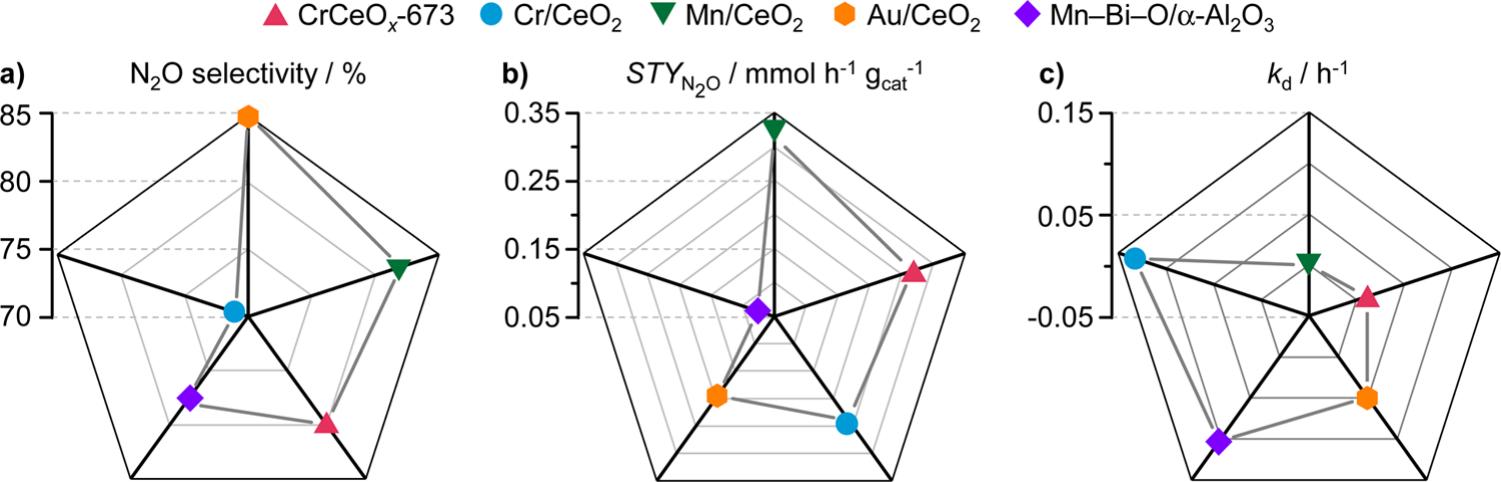
Performance comparison of the best catalysts presented in this work with the benchmark systems, in terms of (a) highest achieved N_2_O selectivity, (b) STY of N_2_O, and (c) deactivation constant (*k*_d_). *k*_d_ value was obtained by fitting the normalized rate of N_2_O production over 50+ h on stream to a power function of the form *y* = *ax*^–*k*_d_^. Reaction conditions: *T*_bed_ = 673 K; *m*_cat_ = 0.01–0.2 g; GHSV = 15,000 (a) or 450,000 cm^3^ h^–1^ g_cat_^–1^(b, c); feed composition = 8 vol % NH_3_, 8 vol % O_2_, 4 vol % Ar, 80 vol % He; *P* = 1 bar. The N_2_O selectivity of Au/CeO_2_ and Mn–Bi–O/α-Al_2_O_3_ was evaluated at 573 and 623 K, respectively.

Having developed promising catalytic systems (i.e., Cr/CeO_2_ and CrCeO_*x*_-673), we sought to evaluate how they compare to previously reported benchmark systems, comprising Au nanoparticles supported on CeO_2_ (Au/CeO_2_), Mn single atoms stabilized on the surface of CeO_2_ (Mn/CeO_2_) and a mixed manganese–bismuth oxide supported on alumina (Mn–Bi–O/α-Al_2_O_3_). The comparison was made based on several performance metrics, namely, the highest achieved N_2_O selectivity, STY_N2O_, and catalyst stability (Figure 7). Although CrCeO_*x*_-673 was slightly inferior to Au/CeO_2_ in terms of N_2_O selectivity (Figure 7a), it was comparable with Mn/CeO_2_ and superior to all others. Furthermore, it displayed N_2_O productivity second only to that of Mn/CeO_2_, which exceeded it by a narrow margin (Figure 7b). Finally, the excellent stability of CrCeO_*x*_-673 also made it the sole competitor to Mn/CeO_2_, whereas all other catalysts experienced deactivation (Figure 7c). In contrast to CrCeO_*x*_-673, Cr/CeO_2_ ranks last in all categories except for , which further highlights the benefits of establishing a strong interaction between the metal and CeO_2_ by adopting a suitable synthetic technique. Based on this evaluation, CrCeO_*x*_-673 stands out as a highly competitive catalytic system for selective and stable N_2_O production via NH_3_ oxidation.

## Conclusions

Contrary to the conventional wisdom of using manganese as the main component of catalysts for selective NH_3_ oxidation to N_2_O, in this work, we demonstrate the viability of a previously overlooked metal, Cr, for this purpose. We utilized atomic scale engineering to exploit the properties of the metal and the carrier by incorporating a small amount of isolated Cr atoms (1 wt %) within the CeO_2_ crystal through CP. The choice of the synthetic technique was shown to be crucial, as simple impregnation resulted in poorly stabilized atoms of Cr, which agglomerated during the reaction, whereas introducing Cr in the carrier allowed atomic dispersion and the stable rate of N_2_O formation to be maintained for 100 h on stream. The calcination temperature was also found to regulate the extent of the Cr-induced increase in the density of oxygen vacancies in CeO_2_, which, in turn, was identified as a performance descriptor. In addition, the rate of associated surface reduction and reoxidation processes was enhanced, thus resulting in a comprehensive improvement of catalyst oxygen buffer ability. The latter is of particular note, as the combination of kinetic and TAP studies evidenced direct participation of lattice oxygen of CeO_2_ in N_2_O formation. Further mechanistic insights were obtained by DFT simulations, revealing that it is the ability of Cr atoms to dynamically change oxidation state and coordinatively restructure that enables facile oxygen vacancy creation and stabilization of nitroxyl and nitrous acid-like reaction intermediates. Furthermore, reactant adsorption was found to partially occur over Ce atoms, thus bringing us to the conclusion that isolated Cr atoms form a catalytic ensemble with the proximal CeO_2_. Thus, this catalyst serves as an example of isolated metal atoms inducing carrier modification and causing the latter to take on a cocatalytic role, a phenomenon that has only recently begun to be recognized in the SAC community.^68−70^ In fact, the curated partnership of Cr and CeO_2_ has allowed the attainment of catalytic performance that superseded nearly all benchmark systems and was on par with that of the state-of-the-art Mn/CeO_2_. The understanding of the catalyst design principles acquired in this work, whose viability has been demonstrated for an underexplored and generally disregarded metal, will aid in this endeavor and bring us one step closer to the implementation of a more economic and sustainable method of N_2_O synthesis.

## Data Availability

The experimental and computational data presented in the main figures of the manuscript are publicly available through the Zenodo (10.5281/zenodo.8285840) and ioChem-BD (https://iochem-bd.iciq.es/browse/review-collection/100/64626/648b674425655ee479759631) repositories, respectively. Further data supporting the findings of this study are available in the Supporting Information. All other relevant source data are available from the corresponding author upon request.

## Supporting Information Available

Summary of results of elemental analysis and N_2_ sorption experiments; simulated single-atom Cr adsorption and substitution energies on different facets of CeO_2_; simulated adsorption energies of reactants and key intermediates on the substitutional (111)-based CrCeO_*x*_ catalyst model; schematic of the experimental setup for NH_3_ oxidation; additional catalytic tests of Cr-based materials; H_2_-TPR profiles of Cr-based catalysts and corresponding carriers; HAADF-STEM micrographs with corresponding particle size distributions of CrCeO_*x*_-*T* catalysts; the scatter plots of H_2_ consumption in H_2_-TPR tests vs oxygen uptake and N_2_O space-time yield vs apparent activation energy of NH_3_ conversion for Cr-based catalysts; Raman spectra of CeO_2_-based materials; results of the kinetic investigation; HAADF-STEM micrographs and EDX mappings of as-prepared and used Cr-based catalysts; XRD patterns and low-temperature EPR spectra of Cr-based materials; UV–vis spectra of CeO_2_-based catalysts acquired over the course of reduction and reoxidation treatment; library of simulated structures of adsorbed and lattice-substituted single Cr atoms on CeO_2_; simulated process of oxygen cycling enabled by the vacancy in the (111) based CrCeO_*x*_ catalyst; and simulated reaction sequence of N_2_O formation over an adsorbed isolated Cr atom (PDF)
