# Supplementary material for: Lattice-Stabilized Chromium Atoms on Ceria for N2O Synthesis
Source: ACS Catal. 2023 Nov 28;13(24):15977–90. doi: 10.1021/acscatal.3c04463 (PMC10728900; doi:10.1021/acscatal.3c04463)
Supplement: Supplementary file 1 — cs3c04463_si_001.pdf [file cs3c04463_si_001.pdf]

Supporting Information

Lattice-Stabilized Chromium Atoms on Ceria for N_2O Synthesis

*Qingxin Yang[a,‡], Ivan Surin[a,‡], Julian Geiger[b], Henrik Eliasson[c], Mikhail Agrachev[d], Vita A. Kondratenko[e], Anna Zanina[e], Frank Krumeich[f], Gunnar Jeschke[d], Rolf Erni[c], Evgenii V. Kondratenko[e], Núria López[b] and Javier Pérez-Ramírez[a,]**

[a]Institute for Chemical and Bioengineering, Department of Chemistry and Applied Biosciences, ETH Zürich, Vladimir-Prelog-Weg 1, 8093 Zürich, Switzerland

[b]Institute of Chemical Research of Catalonia (ICIQ-CERCA), Av. Països Catalans 16, 43007 Tarragona, Spain

[c]Electron Microscopy Center, Empa - Swiss Federal Laboratories for Materials Science and Technology (EMPA), Überlandstrasse 129, 8600 Dübendorf, Switzerland

[d]Laboratory of Physical Chemistry, Department of Chemistry and Applied Biosciences, ETH Zürich, Vladimir-Prelog-Weg 2, 8093 Zürich, Switzerland

[e]Advanced Methods for Applied Catalysis, Leibniz-Institut für Katalyse e. V., Albert Einstein-Str. 29a, 18059 Rostock, Germany

[f]Laboratory of Inorganic Chemistry, Department of Chemistry and Applied Biosciences, ETH Zürich, Vladimir-Prelog-Weg 1, 8093 Zürich, Switzerland

‡ Equal contribution. * Corresponding author. E-mail: jpr@chem.ethz.ch.

Table S1. Metal content and specific surface area of the catalysts in this work.

Catalyst	Preparation method	Metal content[a] / wt.%	S_{BET}[b] / m^2 g^{-1}
Cr/CeO_2	IWI[c]	0.94	32 (42)[d]
Cr/ZrO_2	IWI	0.75	54 (47)
Cr/Al_2O_3	IWI	0.57	11 (9)
Cr/Nb_2O_5	IWI	0.93	4 (4)
$CrCeO_x$-673	CP[e]	0.91	66 (64)
$CrCeO_x$-873	CP	0.96	31 (31)
$CrCeO_x$-1073	CP	0.68	14 (18)

[a] Determined by ICP-OES; [b] Determined by N_2 sorption; [c] Incipient wetness impregnation; [d] Value in brackets indicates the specific surface area of the Cr-free material. [e] Co-precipitation.

Table S2. Adsorption energies, E_{ads} (in eV) calculated using **Equation 8**, evaluated with PBE + U and HSE03-13 for single-atom chromium adsorbed on common coordination motifs provided by ceria low-index facets. The optimized geometries are presented in **Figure S16**. All values are given in eV.

Cr^{x+}	(100)		(110)		(111)	
	PBE + U	HSE	PBE + U	HSE	PBE + U	HSE
2	-1.41	-1.11	-0.61	+0.78	-	-
3	-1.03	1.35	-	-	-1.08	0.93
4	-	-	-2.48	-0.13	-1.24	0.47

Table S3. Substitutional energies, E_{sub}, and metal oxidation states, OS, of single-atom chromium replacing a surface cerium atom evaluated with PBE + U for two different setups of the Hubbard U-correction during structural optimization: (*i*) PBE + U(Ce): 4.5 eV on Ce, and (*ii*) PBE + U(Ce, Cr): 4.5 eV on Ce and 3.5 eV on Cr.[1] The substitutional energies were calculated using **Equation 9**. All energy values are given in eV. From the different DFT setups it becomes evident that neglecting the U-correction for substitutional chromium leads to an artificial excess electron localization on cerium centers due to the restructuring (see optimized structures in **Figure S16**), resulting in the assignment of a formal OS of 6+ for the (100) and (111) facets, as well as large energy discrepancies between the two methodologies. Further energy refinement with the HSE03-13 hybrid functional for both setups (denoted HSE (UCe) and HSE (UCeCr), respectively) leads to consistent energies, due to the similarity of the pre-optimized geometries and the accurate description of the electronic structure by the hybrid functional.

DFT setup	(100)		(110)		(111)	
	E_{sub}	OS	E_{sub}	OS	E_{sub}	OS
PBE + U(Ce)	-6.27	6	-5.29	4	-4.63	6
HSE (UCr)	-4.87	6	-5.88	4	-3.38	6
PBE + U(Ce, Cr)	-2.27	4	-2.27	4	-0.49	4
HSE (UCeCr)	-5.13	4	-5.35	4	-3.45	4

Table S4. Adsorption energies of the relevant proposed intermediates *en route* to N_2O formation on the metal-adjacent vacancy at the substitutional (111)-based $CrCeO_x$ catalyst model (as shown in **Figure 6** of the main manuscript).

Adsorbate	PBE + U(Ce)	HSE (UCe)	PBE + U(Ce,Cr)	HSE (UCeCr)
O_2	-0.49	-1.06	-0.40	-0.45
NH_3	-0.80	-0.79	-0.69	-0.19
O_2, NH_3	-1.11	-1.71	-1.00	-1.13
HNO	-1.37	-2.00	-1.26	-1.34
$H_2N_2O_2$	-0.66	-1.63	-1.35	-0.96
H_2O	-0.92	-0.98	-0.79	-0.35

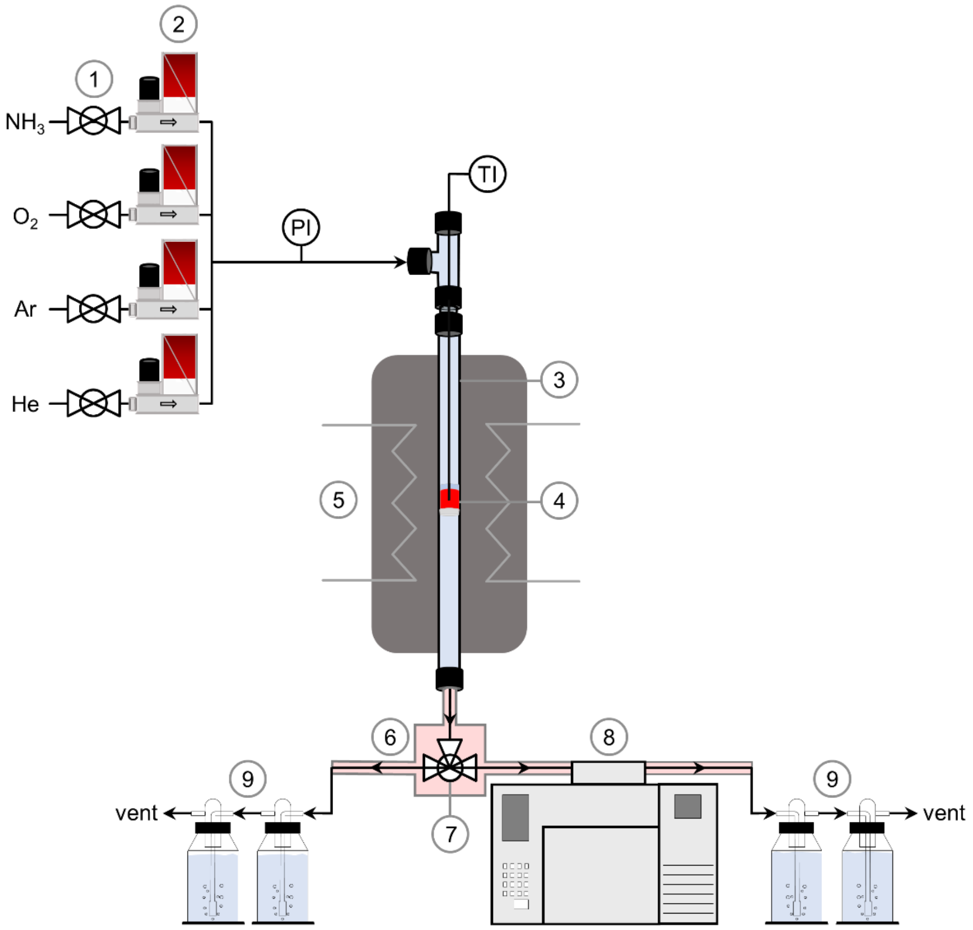

Figure S1. Scheme of the set-up for ammonia oxidation. 1: two-way on/off valves, 2: mass flow controllers, 3: quartz reactor, 4: catalyst bed, 5: oven, 6: heat tracing (red background), 7: three-way sampling valve, 8: gas chromatograph coupled to a mass spectrometer (GC-MS), 9: H_2O and H_2SO_4 scrubbers, PI: pressure indicator, and TI: temperature indicator.

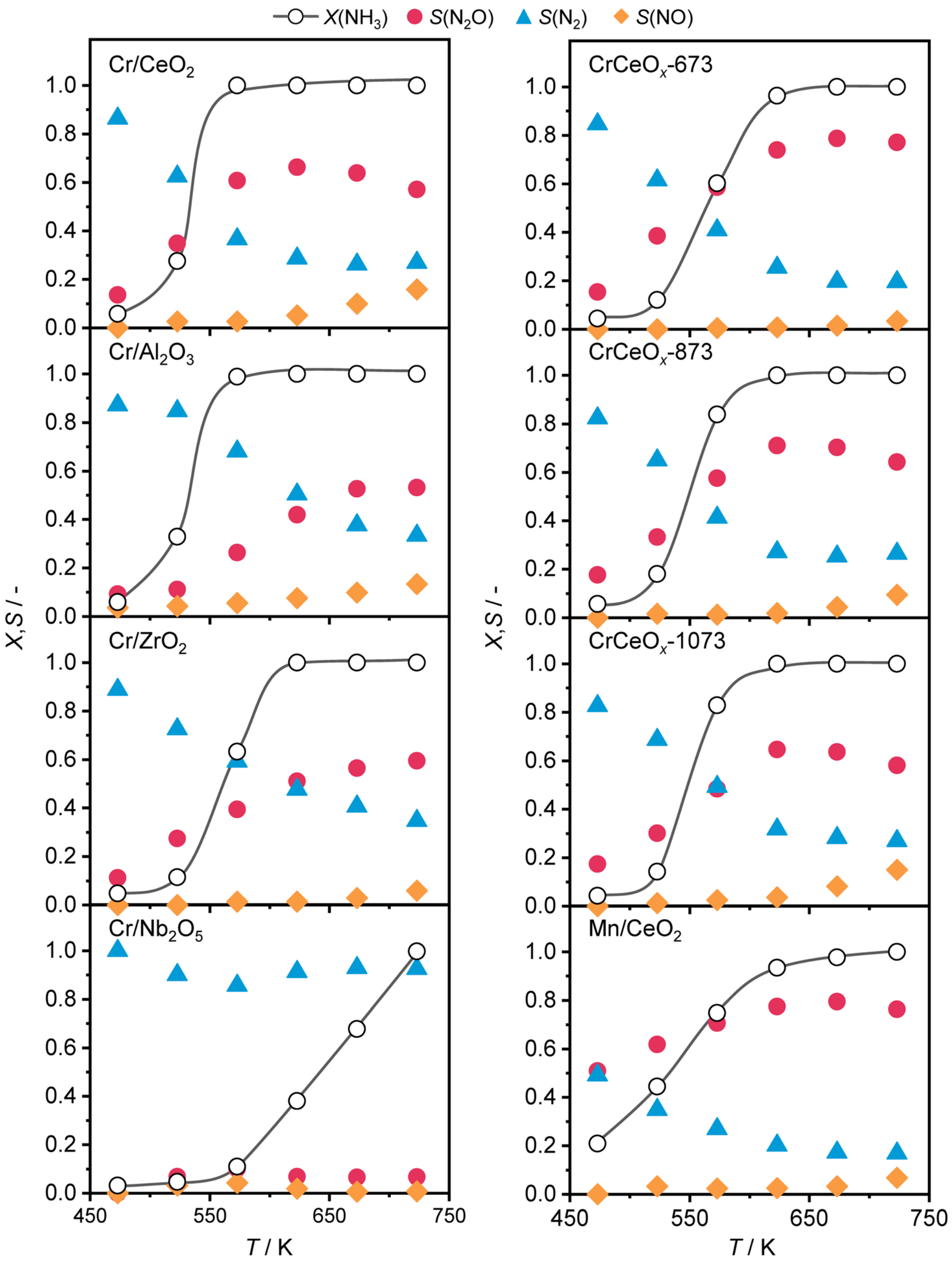

Figure S2. NH_3 conversion and product selectivity of Cr-based catalysts and reference materials as a function of temperature. Reaction conditions: T_{bed} = 473-723 K; m_{cat} = 0.2 g; $GHSV$ = 15,000 cm^3 h^{-1} g_{cat}^{-1}; Feed composition = 8 vol.% NH_3, 8 vol.% O_2, 4 vol.% Ar, 80 vol.% He; P = 1 bar.

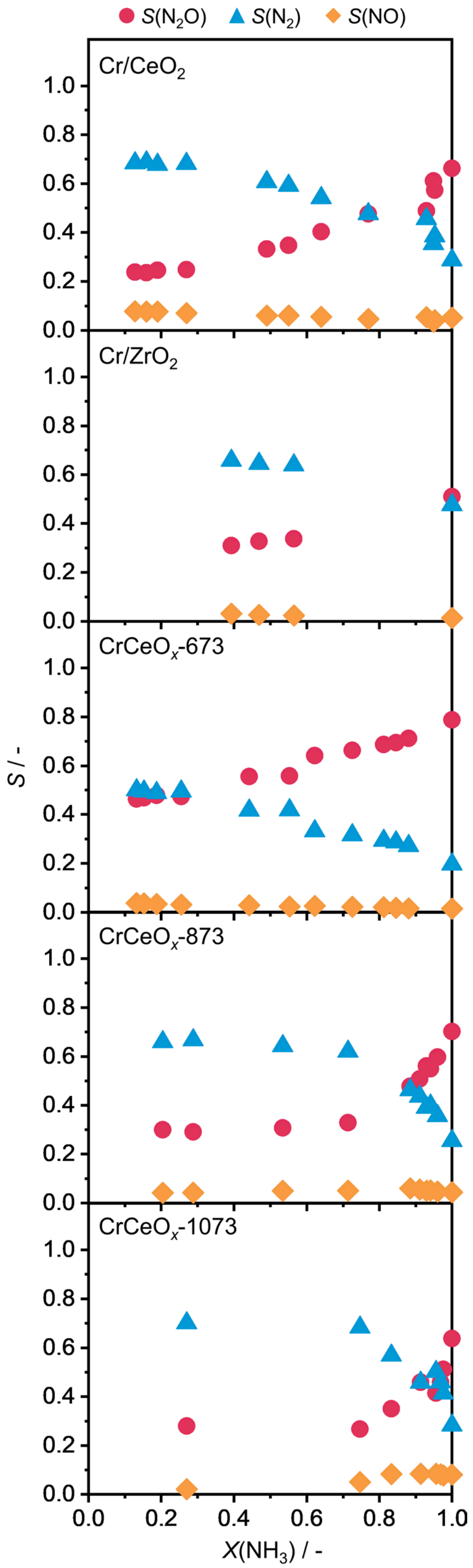

Figure S3. Product selectivity as a function of NH_3 conversion of Cr-based catalysts. Reaction conditions: T_{bed} = 673 K; m_{cat} = 0.002-0.2 g; *GHSV* = 15,000-3,750,000 $cm^3\ h^{-1}\ g_{cat}^{-1}$; Feed composition = 8 vol.% NH_3, 8 vol.% O_2, 4 vol.% Ar, 80 vol.% He; P = 1 bar.

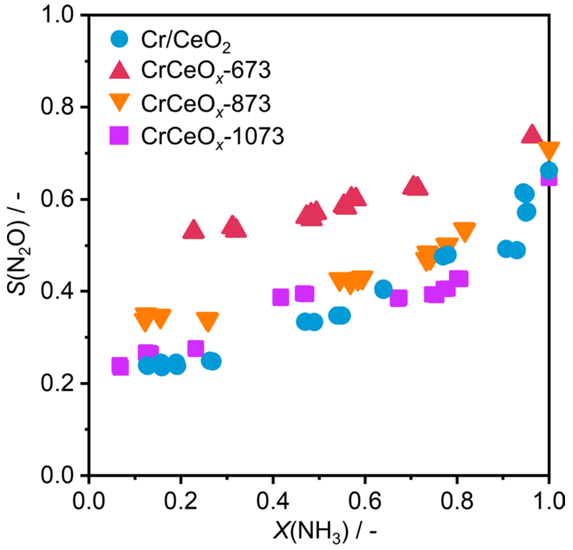

Figure S4. N_2O selectivity of $CrCeO_x$-*T* and Cr/CeO_2 as a function of NH_3 conversion. Reaction conditions: T_{bed} = 673 K; m_{cat} = 0.2-0.002 g; *GHSV* = 15,000-3,750,000 $cm^3\ h^{-1}\ g_{cat}^{-1}$; Feed composition = 8 vol.% NH_3, 8 vol.% O_2, 4 vol.% Ar, 80 vol.% He; *P* = 1 bar.

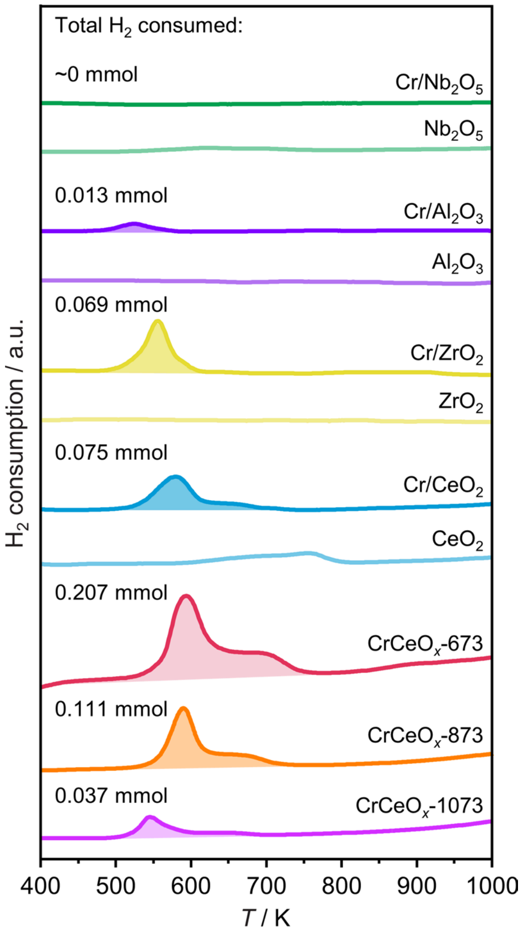

Figure S5. H_2-TPR profiles of as-prepared Cr-based catalysts and corresponding supports. The absence of a reduction peak in the profile of Cr/Nb_2O_5 is likely the result of the formation of a highly stable $CrNbO_4$ phase.[2] The amount of H_2 consumed was quantified based on a calibration using a known quantity of CuO.

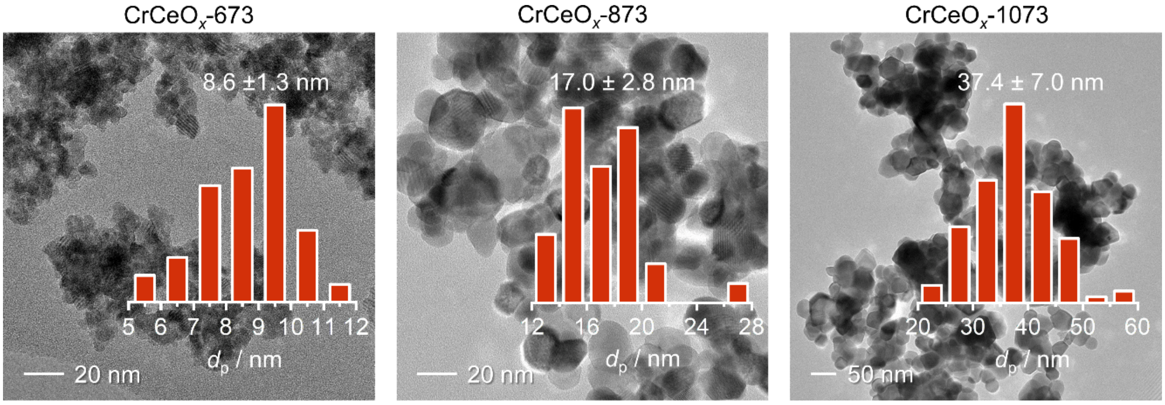

Figure S6. TEM micrographs with corresponding size distributions of ceria particles of as-prepared $CrCeO_x$-*T* catalysts.

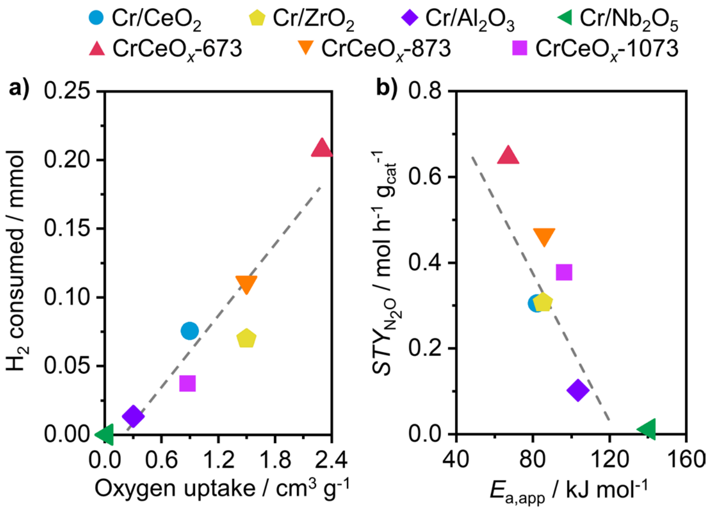

Figure S7. a) Total amount of H_2 consumed in H_2-TPR experiment (**Figure S5**), quantified by integrating the area of the reduction peak, as a function of oxygen uptake and **b)** STY_{N2O} as a function of apparent activation energy of NH_3 oxidation, $E_{a,app}$, of Cr-based catalysts. Reaction conditions: T_{bed} = 673 K; m_{cat} = 0.002-0.2 g; $GHSV$ = 15,000-3,000,000 cm^3 h^{-1} g_{cat}^{-1}; Feed composition = 8 vol.% NH_3, 8 vol.% O_2, 4 vol.% Ar, 80 vol.% He; P = 1 bar.

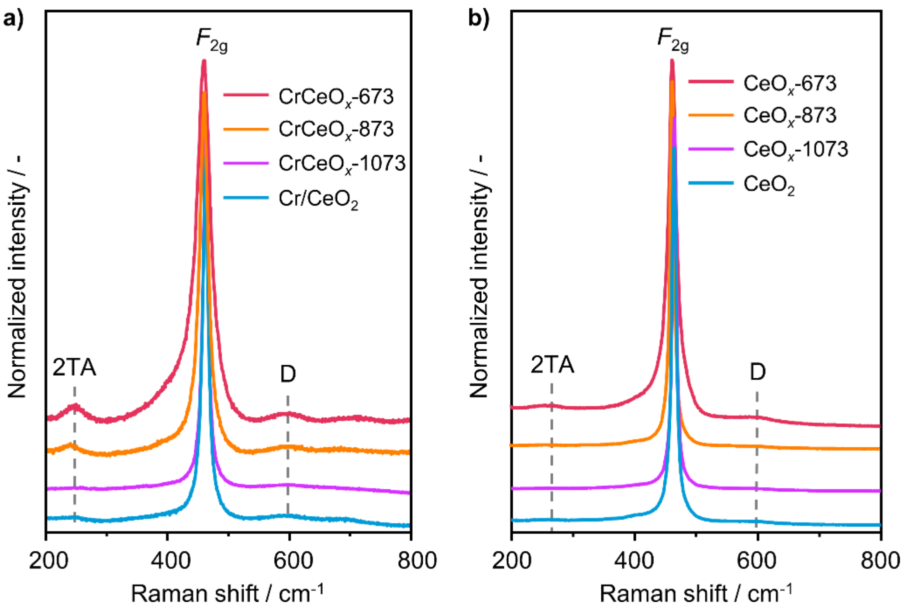

Figure S8. Raman spectra of **a)** as-prepared catalysts and **b)** Cr-free reference materials. Raman band at 462 cm^{-1} is characteristic of F_{2g} mode of CeO_2 with cubic fluorite-type structure.[3] Signal at 595 cm^{-1} is characteristic of oxygen defect-induced (D) mode.[3,4] The Raman band at 250 cm^{-1} belongs to the second-order transverse acoustic (2TA) mode, characteristic of small CeO_2 nanocrystals.[4] In addition to the contribution of 2TA mode to Raman band at 250 cm^{-1}, recent studies state that this feature could be related to oxygen defects.[5,6] Generally, the intensity of D- and 2TA-mode signals increases upon introduction of Cr, indicative of the metal facilitating the formation of oxygen vacancies. Similarly, the signal intensity increases with as the temperature of calcination of $CrCeO_x$-T catalysts is reduced, representative of a larger concentration of oxygen vacancies.

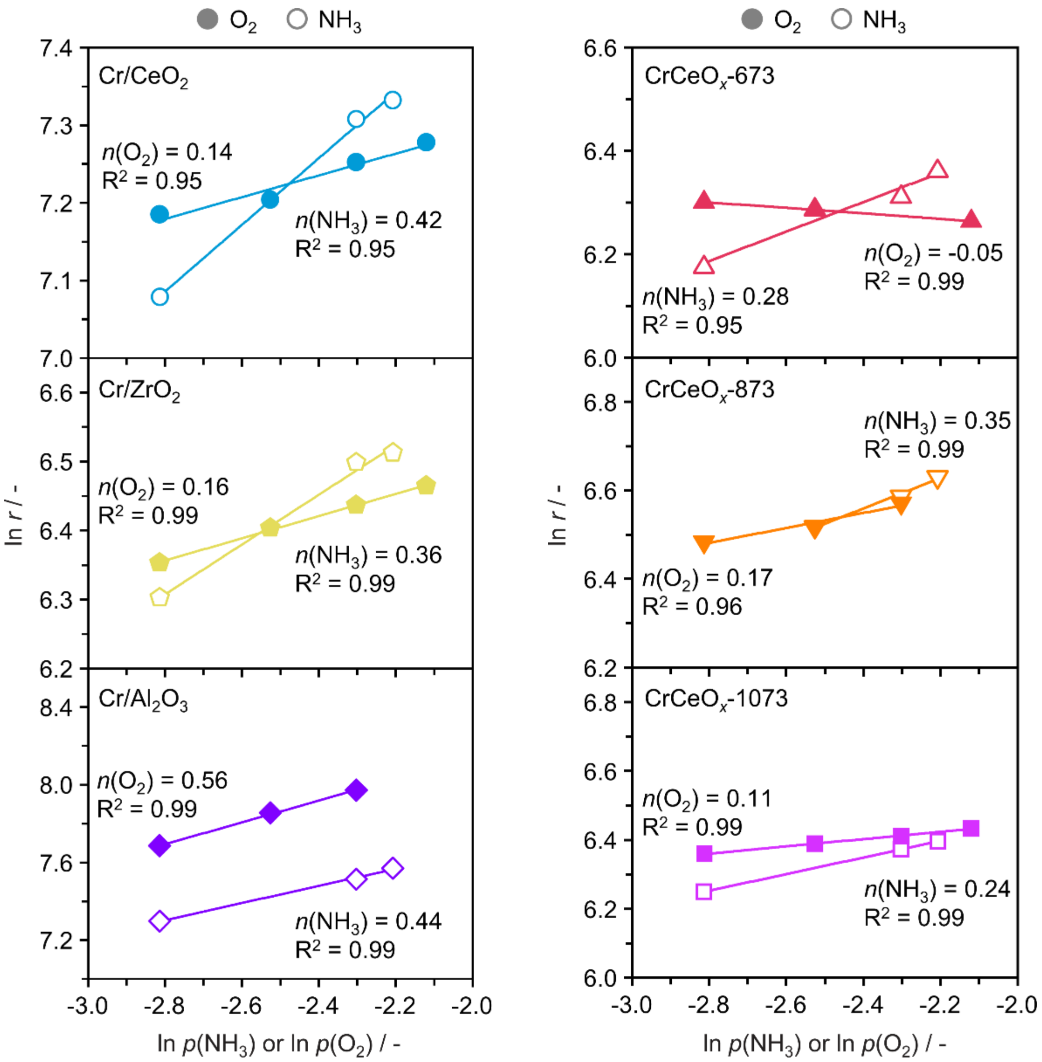

Figure S9. Rates of NH_3 conversion as a function of inlet partial pressure of NH_3 and O_2. The partial reaction orders, n_i, and quality of the fit are indicated. Reaction conditions: $T_{bed} = 673$ K, $m_{cat} = 0.002\text{-}0.03$ g, $GHSV = 200{,}000\text{-}3{,}000{,}000$ cm^3 h^{-1} g_{cat}^{-1}; Feed composition = 6-11 vol.% NH_3, 6-12 vol.% O_2, 4 vol.% Ar, 76-82 vol.% He; $P = 1$ bar.

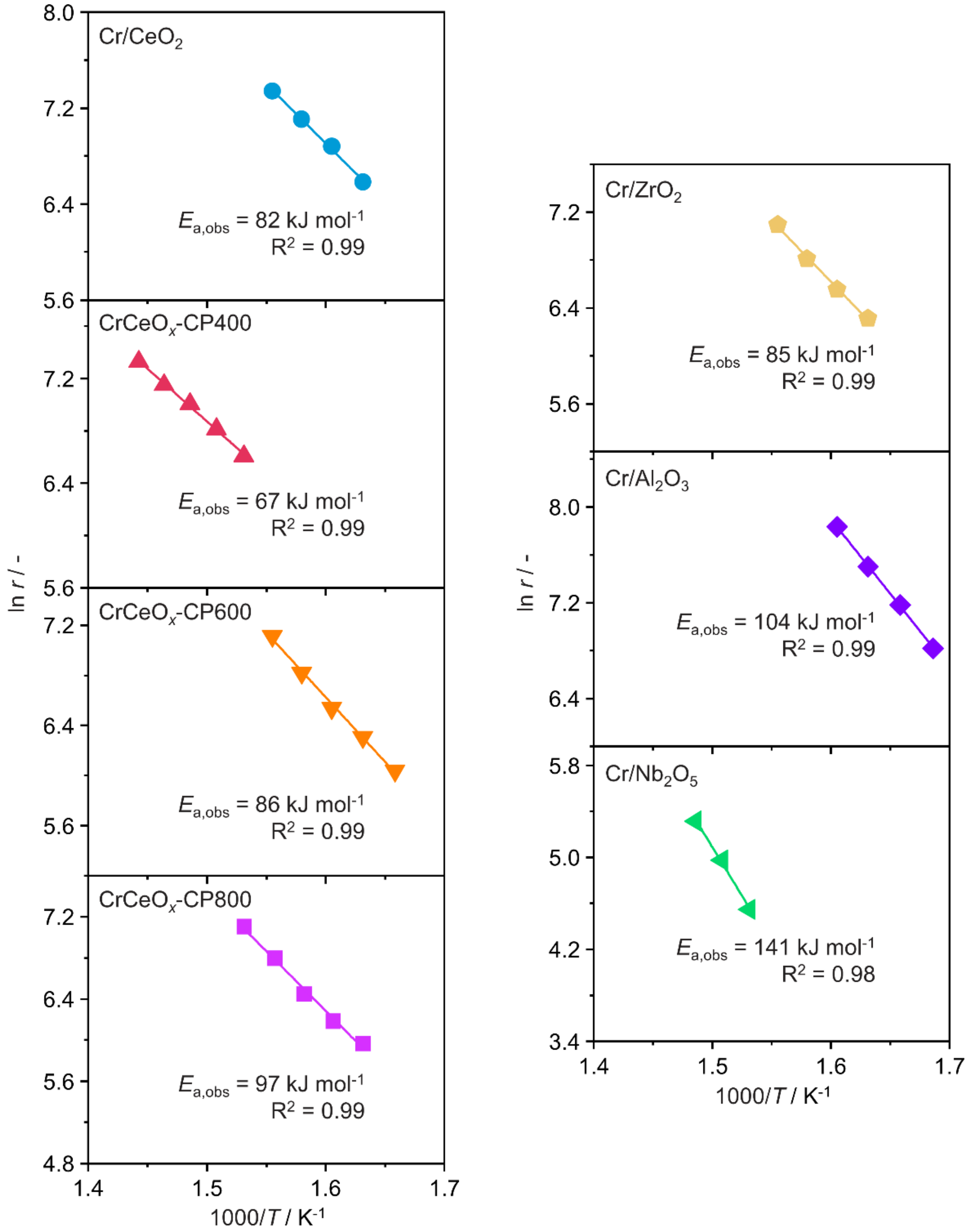

Figure S10. Arrhenius plots and calculated apparent activation energies of NH_3 conversion ($E_{a,obs}$). Reaction conditions: T_{bed} = 653-693 K, m_{cat} = 0.002-0.03 g, $GHSV$ = 200,000-3,000,000 cm^3 h^{-1} g_{cat}^{-1}; Feed composition = 8 vol.% NH_3, 8 vol.% O_2, 4 vol.% Ar, 80 vol.% He; P = 1 bar.

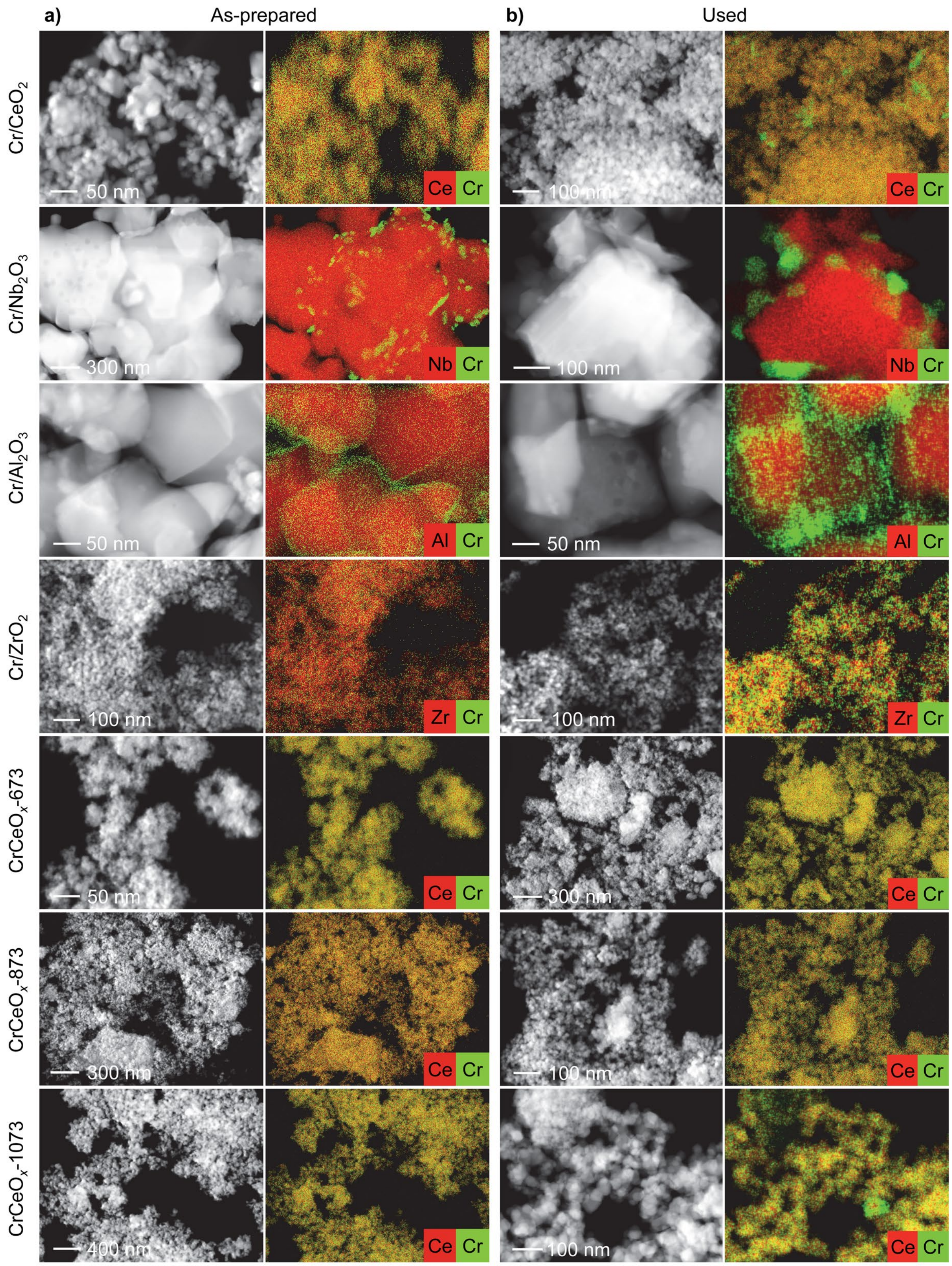

Figure S11. HAADF-STEM micrographs with EDX maps of **a)** as-prepared and **b)** used Cr-based catalysts.

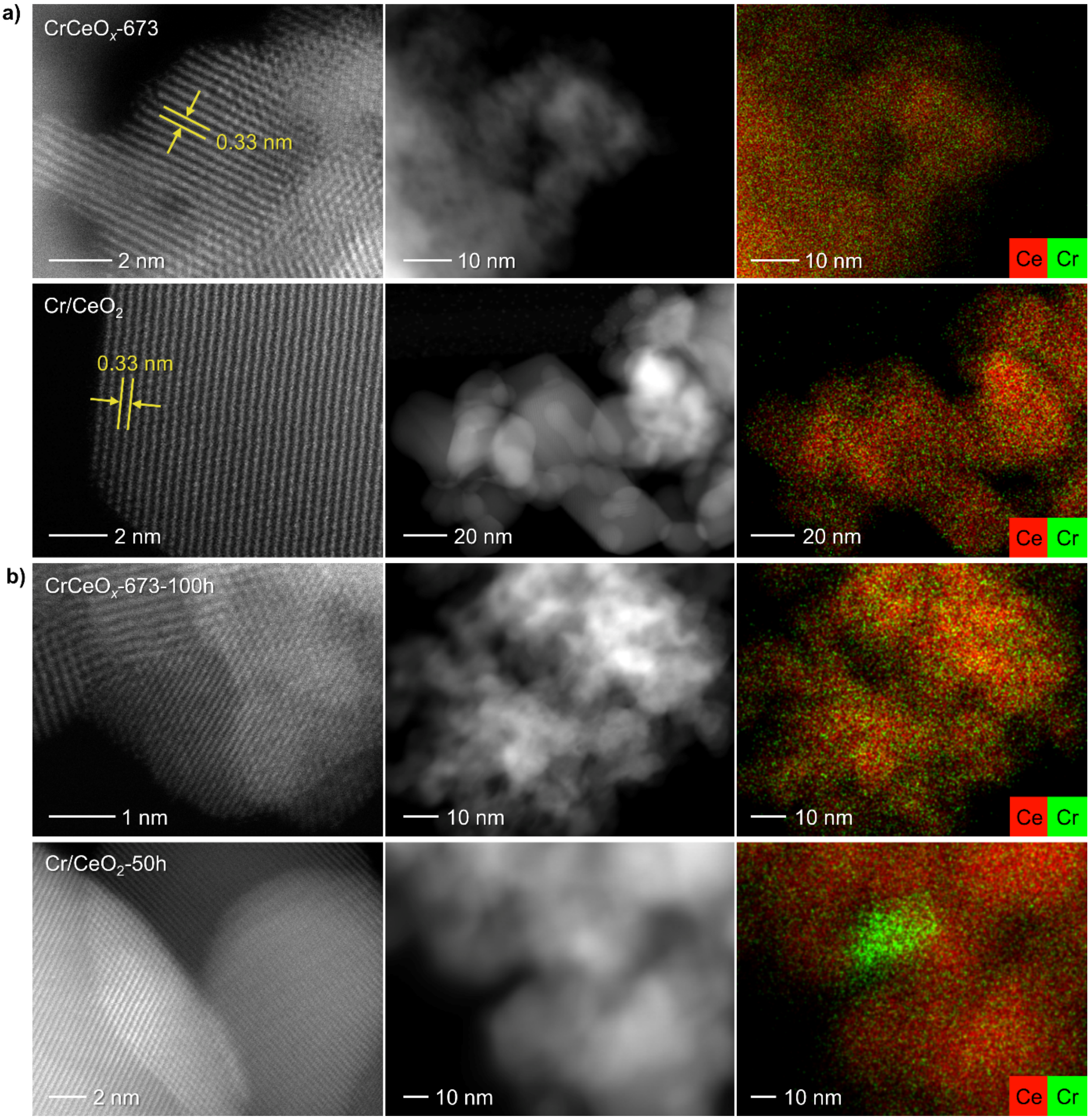

Figure S12. HAADF-HRSTEM images with EDX maps of **a)** as-prepared and **b)** used $CrCeO_x$-673 and Cr/CeO_2 catalysts. In **a)**, the measured interplanar distance of 0.33 nm between lattice fringes of CeO_2 is consistent with that of (111) planes.

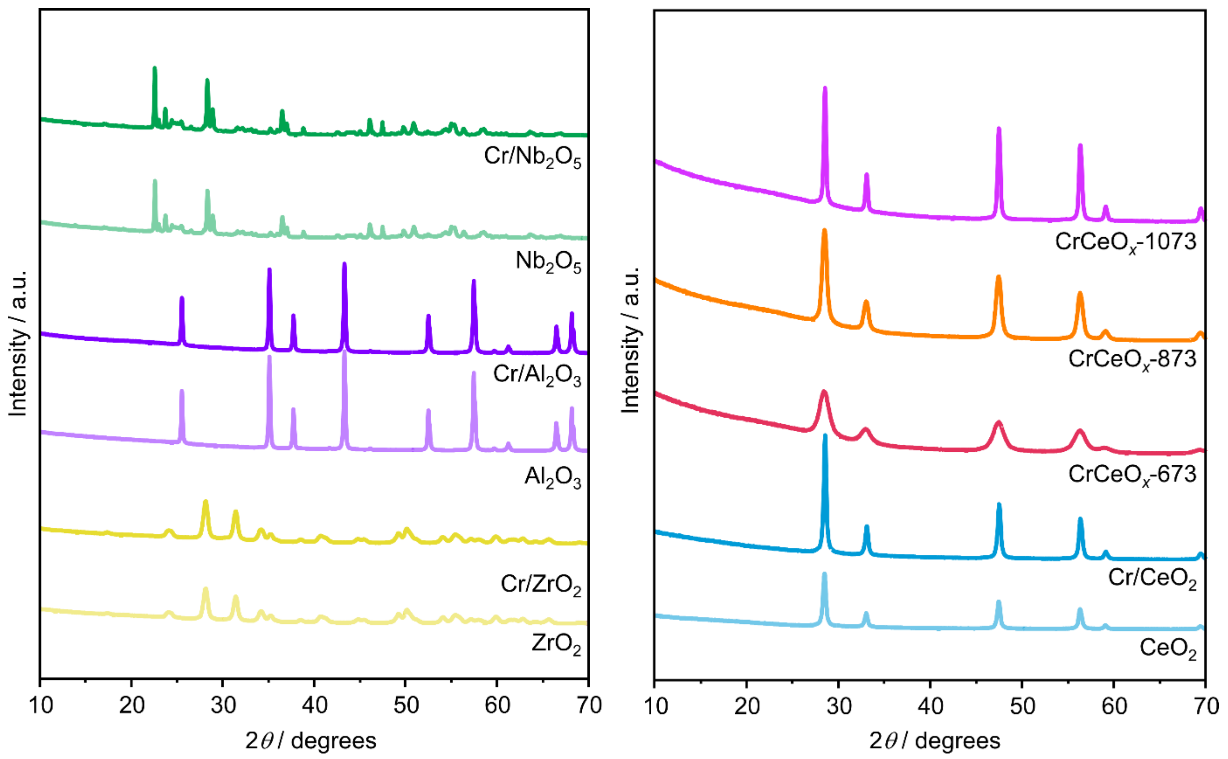

Figure S13. X-ray diffraction patterns of as-prepared Cr-based catalysts and corresponding supports.

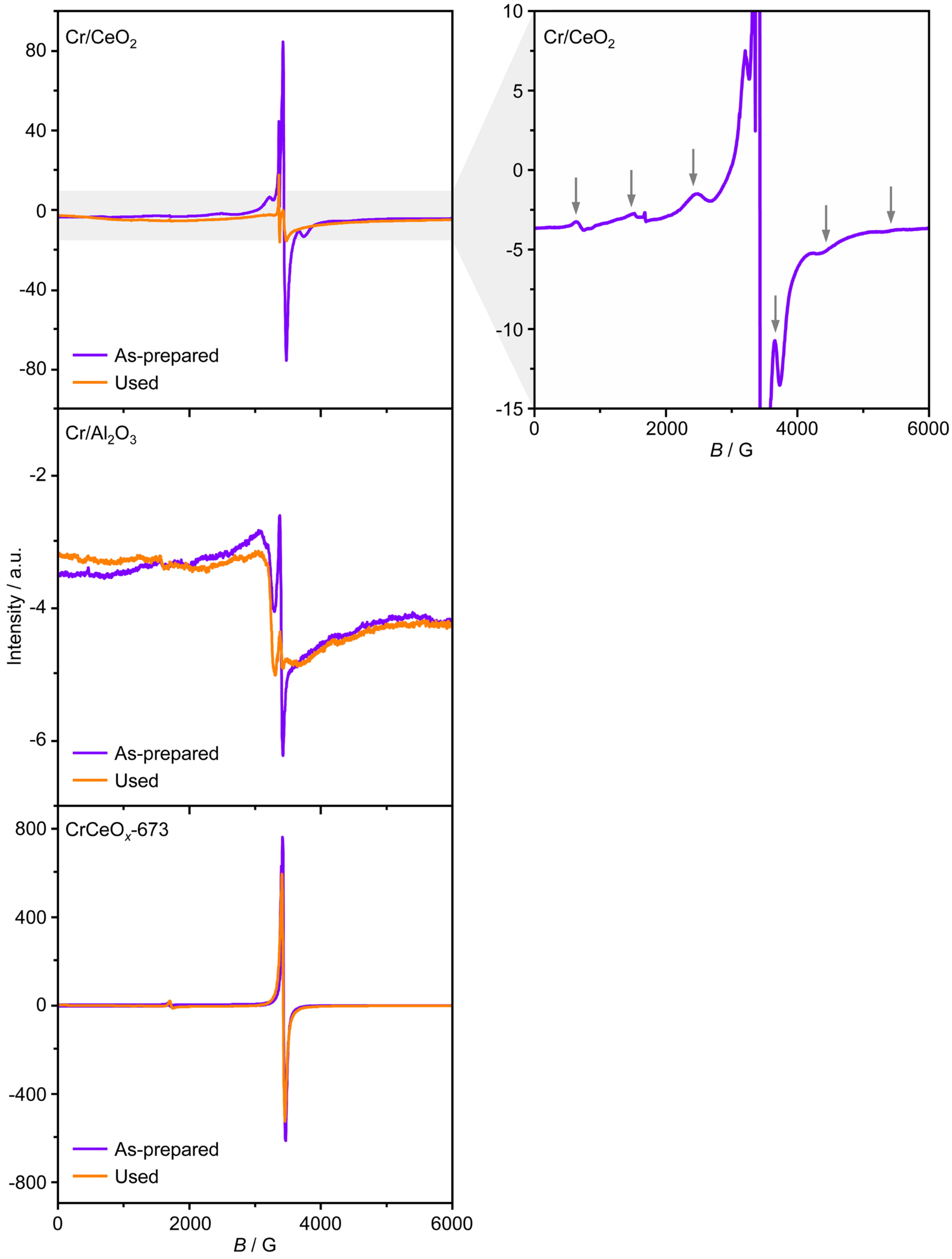

Figure S14. EPR spectra acquired at 10 K of selected as-prepared and used Cr-based catalysts. Grey arrows in the magnified spectrum of Cr/CeO_2 indicate the series of broad peaks due to spin-orbit coupling of Cr.

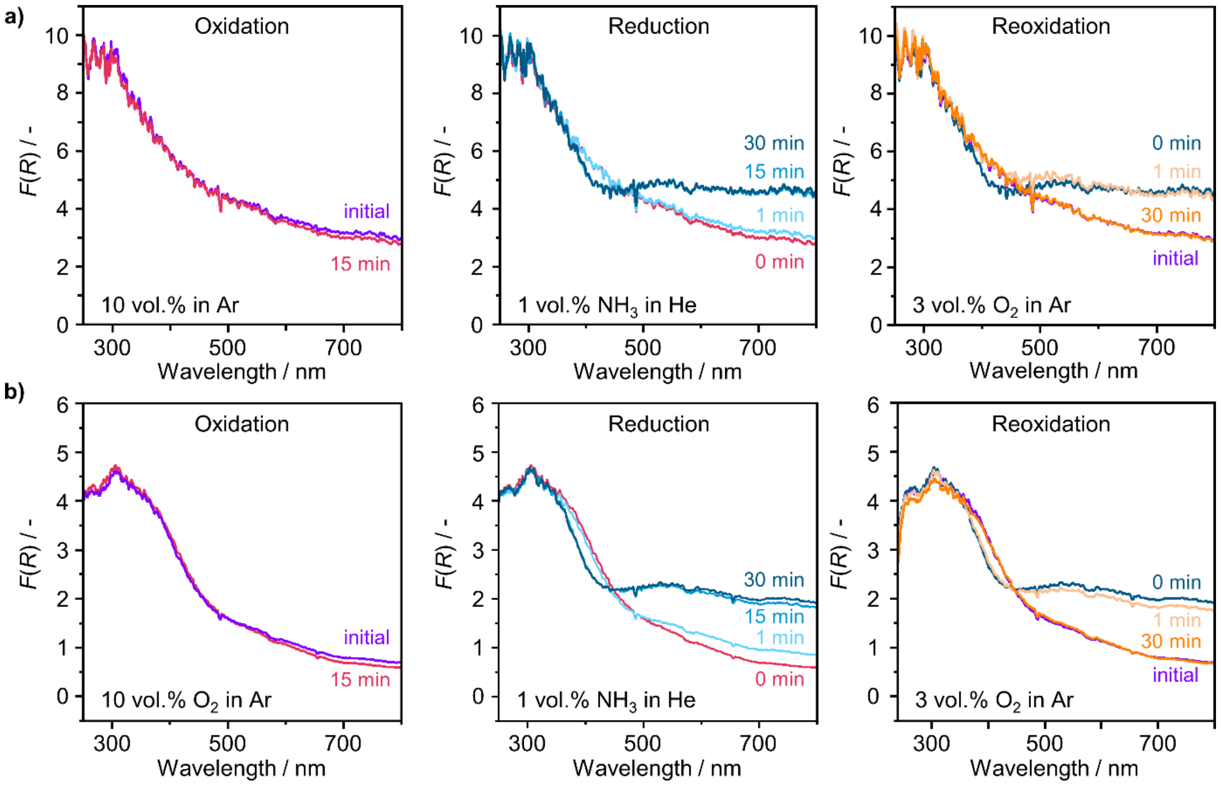

Figure S15. In situ UV-vis spectra of **a)** Cr/CeO_2 and **b)** $CrCeO_x$-673 acquired during initial surface oxidation (left panel), reduction by NH_3 (middle panel), and reoxidation by O_2 (right panel) at 673 K.

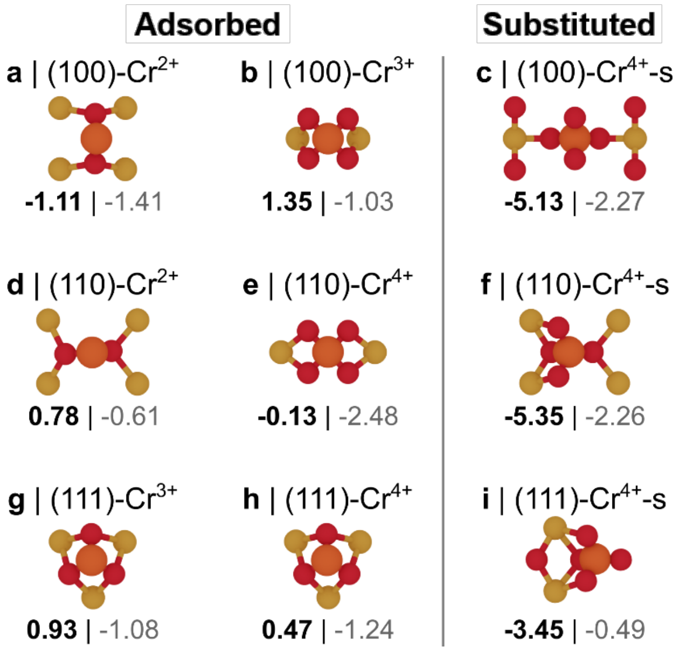

Figure S16. Coordination environments for the catalyst library of single-atom Cr adsorbed on and substituted in (denoted *via* "s") low-index ceria facets. All energies are given in eV and were evaluated with PBE+U (grey) and HSE03-13 (bold).

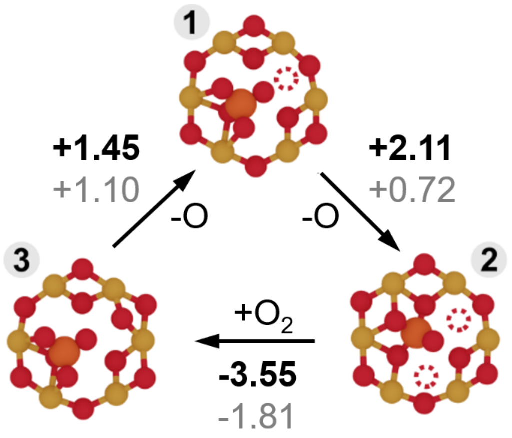

Figure S17. Oxygen cycling enabled by the vacancy in the restructured (111)-based $CrCeO_x$ catalyst (1). Removal of the Cr bound oxygen leads to another restructuring (2), in which the SA-Cr moves further inside the bulk of the material, exposing two surface vacancies. Healing by O_2 restores both vacancies (3) and is therefore highly exothermic. All energies are given in eV and were evaluated with PBE+U (grey) and HSE03-13 (bold).

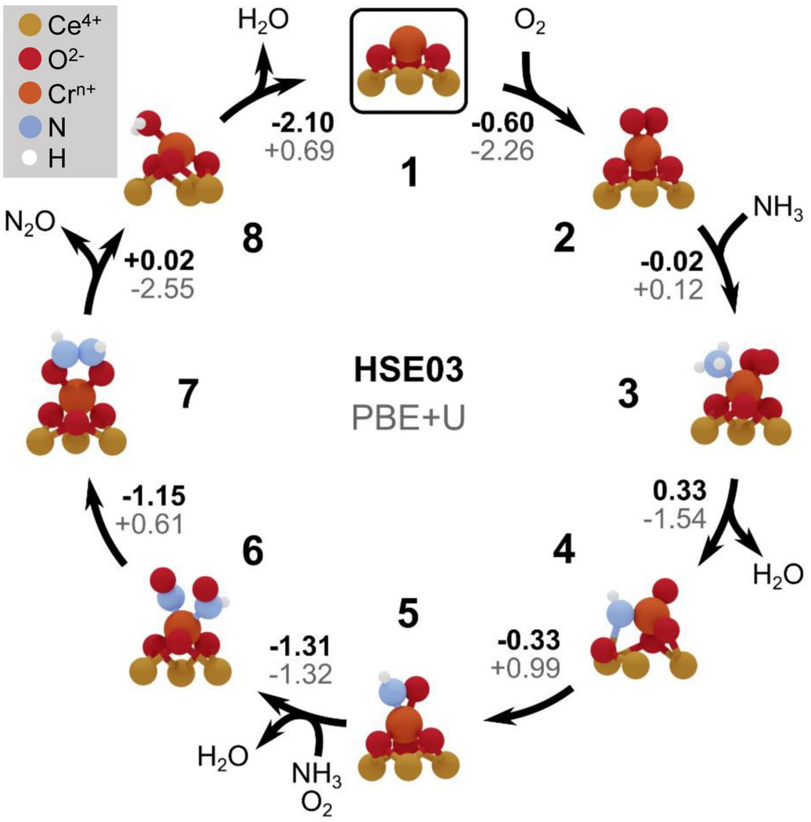

Figure S18. Proposed reaction pathway proceeding *via* nitroxyl (HNO) and cis-hyponitrous acid ($H_2N_2O_2$) intermediates for the most stable catalyst structure of adsorbed Cr^{3+} on the most abundant exposed (111) facet. All energy values are given in eV and were evaluated with PBE+U (grey) and HSE03-13 (bold).

Supporting references

(1) Wang, J.; Gong, X.-Q. A DFT+U study of V, Cr and Mn doped CeO_2(111). *Appl. Surf. Sci.* **2018,** *428*, 377-384, doi:10.1016/j.apsusc.2017.09.120.

(2) Pérez Flores, J. C.; García-Alvarado, F. Electrical conductivity of the oxygen-deficient rutile $CrNbO_{4-\delta}$. *Solid State Sci.* **2009,** *11*, 207-213, doi:10.1016/j.solidstatesciences.2008.05.009.

(3) Loridant, S. Raman spectroscopy as a powerful tool to characterize ceria-based catalysts. *Catal. Today* **2021,** *373*, 98-111, doi:10.1016/j.cattod.2020.03.044.

(4) Wu, Z.; Li, M.; Howe, J.; Meyer, H. M., III; Overbury, S. H. Probing defect sites on CeO_2 nanocrystals with well-defined surface planes by Raman spectroscopy and O_2 adsorption. *Langmuir* **2010,** *26*, 16595-16606, doi:10.1021/la101723w.

(5) Filtschew, A.; Hofmann, K.; Hess, C. Ceria and its defect structure: new insights from a combined spectroscopic approach. *J. Phys. Chem. C* **2016,** *120*, 6694-6703, doi:10.1021/acs.jpcc.6b00959.

(6) Schilling, C.; Hofmann, A.; Hess, C.; Ganduglia-Pirovano, M. V. Raman spectra of polycrystalline CeO_2: a density functional theory study. *J. Phys. Chem. C* **2017,** *121*, 20834-20849, doi:10.1021/acs.jpcc.7b06643.
